# Toward precision medicine using a “digital twin” approach: modeling the onset of disease-specific brain atrophy in individuals with multiple sclerosis

**DOI:** 10.1038/s41598-023-43618-5

**Published:** 2023-09-28

**Authors:** Steven Cen, Mulugeta Gebregziabher, Saeed Moazami, Christina J. Azevedo, Daniel Pelletier

**Affiliations:** 1 Department of Radiology/Neurology, University of Southern California, Los Angeles, USA; 2 Department of Public Health Sciences, Medical University of South Carolina, Charleston, USA; 3 Department of Aerospace and Mechanical Engineering, Viterbi School of Engineering, University of Southern California, Los Angeles, USA; 4 Department of Neurology, Keck School of Medicine, University of Southern California, Los Angeles, USA

**Keywords:** Biomarkers, Medical research, Neurology

## Abstract

Digital Twin (DT) is a novel concept that may bring a paradigm shift for precision medicine. In this study we demonstrate a DT application for estimating the age of onset of disease-specific brain atrophy in individuals with multiple sclerosis (MS) using brain MRI. We first augmented longitudinal data from a well-fitted spline model derived from a large cross-sectional normal aging data. Then we compared different mixed spline models through both simulated and real-life data and identified the mixed spline model with the best fit. Using the appropriate covariate structure selected from 52 different candidate structures, we augmented the thalamic atrophy trajectory over the lifespan for each individual MS patient and a corresponding hypothetical twin with normal aging. Theoretically, the age at which the brain atrophy trajectory of an MS patient deviates from the trajectory of their hypothetical healthy twin can be considered as the onset of progressive brain tissue loss. With a tenfold cross validation procedure through 1000 bootstrapping samples, we found the onset age of progressive brain tissue loss was, on average, 5–6 years prior to clinical symptom onset. Our novel approach also discovered two clear patterns of patient clusters: earlier onset versus simultaneous onset of brain atrophy.

## Introduction

The Digital Twin (DT) concept was first introduced in 2002 as a Fourth Industrial Revolution (Industry4.0) solution to manufacturing intelligence^1^. It was later brought into the medical field as a potential solution for precision medicine, the so-called Health Digital Twin (HDT). In the context of precision medicine, the HDT approach can be defined as “virtual mirror of ourselves that allows us to simulate our personal medical history and state of health using data-driven analytical algorithms and theory-driven physical knowledge”^2^. Recently, HDT has been applied in multiple disease areas, such as oncology^3–5^, geriatrics^6^, cardiology^7–12^, infectious disease^13^, genomic medicine^14^, neurodegenerative diseases^15,16^, vascular medicine^17^, and mental health^18^. The applications of HDT include patient safety, wellbeing management, and health care decision support^19^. Most of the published literature is oriented to system design or concept illustration. There are no standardized methodologies for HDT to date^20,21^. The implementation of HDT includes several major components such as personal devices, AI algorithms, right data to train the AI, and Internet of Things (IoT) for rapid data synchronization while providing real time decision-making^22^.

Towards this AI based solution, identifying appropriate statistical models for specific data structures is critical. In this study we demonstrate an application for modeling neurodegeneration data, specifically brain atrophy data measured by magnetic resonance imaging (MRI) in multiple sclerosis (MS) individuals. For a chronic disease like MS, we must realize that neurodegeneration is a long process occurring over decades^15^. The decision-making for patient care such as drug selection should be based on the entire disease course and not only over a short period of time^23,24^. Moreover, the response to treatment may take years to observe. Therefore, the HDT for a patient with neurodegenerative disease must cover the entire disease-span or even lifespan of patients.

MS is a chronic, immune-mediated, inflammatory and neurodegenerative disorder of the central nervous system and the most common cause of nontraumatic neurologic disability in young adults, affecting over 1,000,000 people in the U.S. and 2.8 million worldwide^25^. Clinically, the diagnosis is defined by the presence of typical neurological symptoms and demyelinating-appearing white matter lesions MRI^26^. Brain atrophy is considered a fundamental aspect of MS, occurring about 3 × faster in MS patients than in healthy controls and likely representing the net accumulation of tissue damage due to the disease^27^. As a major relay nucleus, the thalamus is particularly susceptible to neurodegeneration and has been shown to be one of the earliest regions impacted by atrophy in MS^28,29^. Interestingly, MS plaques and thalamic atrophy can be observed on MRI several years before the onset of first clinical symptoms^28,30^, suggesting that the biological onset of the disease may precede the clinical onset by several years. As such, the ‘true’ biological onset of MS remains unknown. This represents a major barrier to understanding the earliest events in the MS pathophysiology and even the natural history of MS, which is typically based on clinical disease duration.

Using brain atrophy (and more specifically, thalamic atrophy) is an appealing application of the HDT concept in MS. Brain atrophy occurs as part of normal aging, which has been studied extensively in healthy individuals^31^. In the absence of disease, brain volume trajectories are relatively predictable; in fact, recent work has presented normative brain growth charts across the human lifespan^32^. In principle, healthy brain trajectories can be leveraged to create HDTs for patients with neurologic and psychiatric diseases. In the case of MS, one could estimate when an individual MS patient’s thalamic atrophy trajectory deviates from that of a healthy individual. The onset of progressive brain tissue loss should be closer to the true biological onset of the disease, and may further our fundamental understanding of MS.

Using normalized thalamic volumes (thalamic volume/intracranial volume*1000) from brain MRI images, our main objective was to develop statistical learning models to estimate when the thalamic atrophy trajectory of an MS patient deviated from their expected thalamic atrophy trajectory based on their corresponding HDT. The age when MS atrophy trajectory departed from normal aging was defined as the onset of progressive brain tissue loss. We hypothesized that the age of progressive brain tissue loss would be statistically earlier than the age of clinical onset determined by clinicians.

## Challenges

The first challenge is to identify a large longitudinal normal aging dataset with subjects imaged across several decades using brain MRI scans to create the HDT as a normal aging reference for a given MS patient. However, such a longitudinal dataset rarely exists. Most of the datasets collected to study normal aging are cross-sectional. As such, it is difficult to gather real-life datasets to generate reliable trajectories over the entire lifespan.

Even if we had repeated scans for both MS and normal aging, the best statistical method to fit an accurate trajectory curve has not yet been identified. Moreover, it has been shown that the aging brain trajectory is not linear^32–39^. The conventional statistical approach for longitudinal data is a mixed model using year(s) at study entry as the time unit to fit a linear or quadratic trend. However, linear or quadratic approaches may not be the most effective method for representing the complexity of aging data^33,40^, as they can result in biased estimates and low power in statistical tests^40^. On the other hand, as a nonparametric method, a spline model is recommended for its flexibility and robustness to accurately model the age trajectories of neuroimaging markers^39^. There remain several unanswered questions, including whether it is possible to augment longitudinal data from cross-sectional data, whether lifespan data can be augmented from only a few longitudinal data points, how to choose an appropriate spline setting from many mixed spline candidates, and how to select covariates having two-way or three-way interactions with the spline slope.

## Study design

### Overall study design and concept

**Figure 1:**
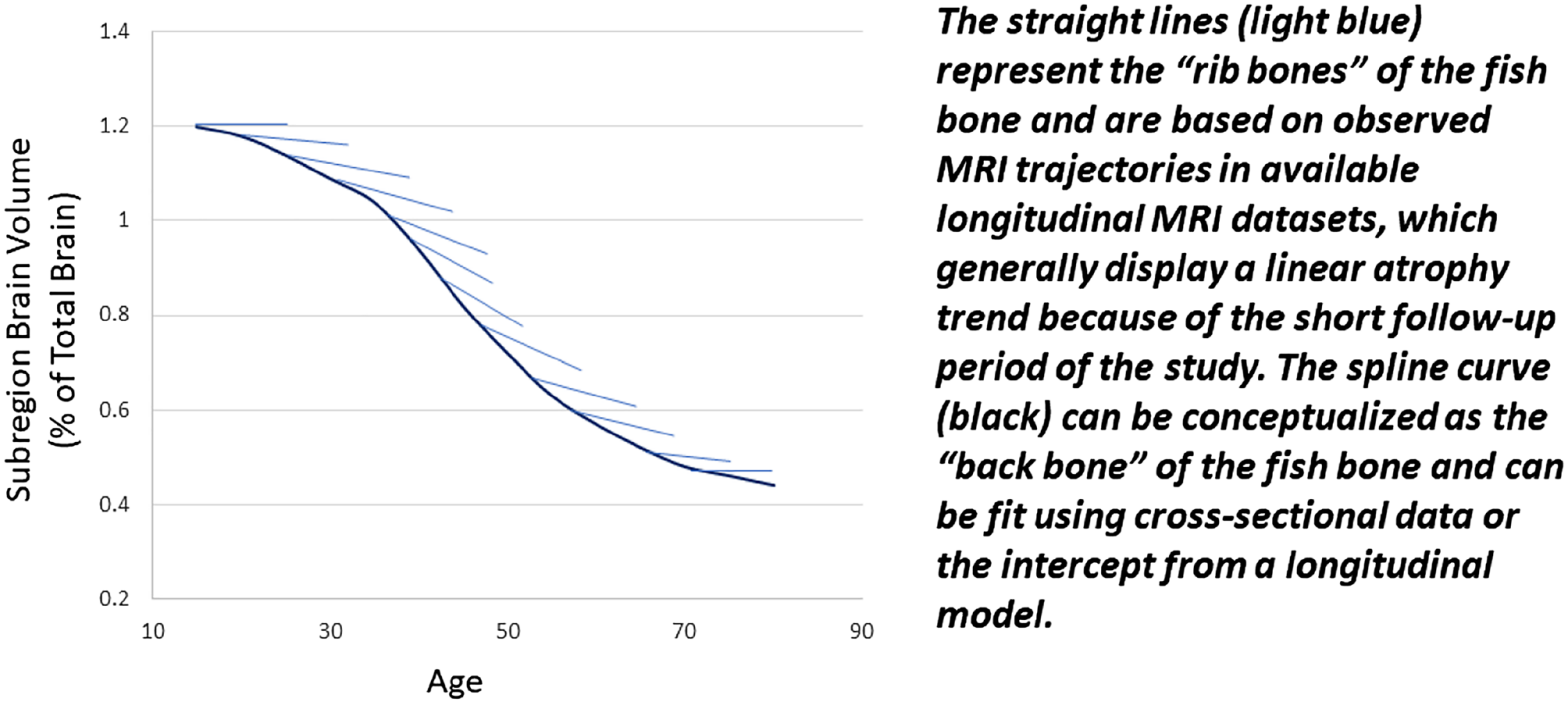
Concept diagram for “fish bone” data structure. A High-resolution version of this Figure can be found in Supplementary Information 2.

Most longitudinal MRI datasets only cover a few years of an individual’s lifespan. For such a short period, when using years of follow-up as the time variable, a linear trend may be the best fit to the data, even though true brain atrophy over lifespan is non-linear. However, when using the actual age in years as the time variable, the model will look very different. For an entire sample, age has a wide coverage for the lifespan, but for each individual, age only covers a small fragment of the lifespan. This data structure can be conceptualized as a “fish bone” (Fig. 1), where the constructed spline curve can be considered the “back bone” and the straight lines (representing observed longitudinal data) can be considered “rib bones”. By using cross-sectional data or the intercept from a longitudinal model with age as the time variable, we should be able to construct the “back bone" of the spline. Adding the “rib bones” from large number of individuals in different age categories can enhance the shape of the spline.

Generally speaking, to obtain the lifespan trajectory, we should observe the “rib bones” in our typical longitudinal datasets and then attempt to construct the “back bone.” In this work, we attempt the converse; using the “back bone”, we attempt to grow the “rib bones”. In other words, our approach is to first fit an accurate spline model from cross-sectional data to model the non-linear trajectory across the lifespan. We then use the age slope to augment the longitudinal data for each normal aging subject, given that a linear model can suffice to model brain atrophy over a short (5-year) period of time.

The study design includes the following steps: (1) identify a well-fitted spline model using cross-sectional data from normal aging populations; (2) augment longitudinal data from this well-fitted spline model using a linear slope at a given age point; (3) compare 12 different mixed spline models through simulated data and identify the mixed spline model that fits the “fish bone” data structure; (4) combine augmented longitudinal normal aging data with longitudinal MS data to fit mixed spline model and compare across 12 mixed spline models; (5) use a manual forward then backward model building strategy to select the covariates from 52 covariate structures; (6) identify the individual age of onset of progressive brain tissue loss with associated 95% confidence interval using a tenfold cross validation procedure through 1000 bootstrapping samples.

### Study sample

Our dataset was assembled from the following three sources (Table 1): (1) The Human Connectome Project (HCP: http://www.humanconnectome.org), (2) Alzheimer’s Disease Neuroimaging Initiative (ADNI: http://www.adni-info.org) and (3) a single-center, prospective cohort MS study conducted from January 2005 through December 2010^29^. Normal aging samples were from HCP, ADNI and 89 healthy control cases from the single-center study. Age at scan date and sex were extracted from each of the data sources. Healthy control subjects (N = 2053) had an overall mean age of 44 ± 21 years (Q1: 27, Q3: 62) with 56% female, while MS subjects (N = 519) had a mean age of 43 ± 10 years (Q1: 36, Q3: 50) with 70% female and an average of 4 ± 1.5 annual scans per subject. Most of the normal aging sample only had one MRI scan, but 228 of them had repeated measures (2.9 ± 1 scans in 2.5 ± 1.4 years). Subjects with age > 90 or < 16 (to avoid brain growth confounding) were excluded.

**Table 1:** Demographic distribution of health control cohorts.

Dataset	# of Subjects	# of MRI time points	Age (Mean ± SD)	Age range	% Female
HCP-D	178	178	19 ± 2	16–22	55.1
HCP	865	865	29 ± 4	22–37	56.3
HCP-A	676	676	58 ± 14	36–90	56.8
Single Center	87	152	40 ± 11	22–65	67.8
ADNI	247	614	75 ± 7	56–89	52.2
Total	2053	2485			

3 T/3D T1-weighted volumetric gradient echo images were processed with FreeSurfer v6.0 to extract thalamic and intracranial volumes. Thalamic volumes were normalized by total intracranial volume and multiplied by 1000. Healthy control subjects had normalized thalamic volumes of 9.7 ± 1 (Q1: 9.1, Q3: 10.4) at study entry, while MS subjects had normalized thalamic volumes at study entry of 9.3 ± 1 (Q1: 8.7, Q3:9.9) (Table 2).

**Table 2:** Demographic distribution and disease characteristics of MS cohort (n = 519).

Age at Study Entry (years)		42.7 ± 9.7, 42.2 (35.5 to 50.4)
Sex	Female	364 (70.13%)
	Male	155 (29.87%)
Age of Clinical Onset (years)		33.5 ± 9.1, 33 (27 to 40)
Disease Type	CIS	90 (17.34%)
	RRMS	391 (75.34%)
	SPMS	38 (7.32%)
History of DMT Use at Study Entry	Never Used	212 (40.85%)
	Ever Used	307 (59.15%)
DMT Exposure (years)		2.5 ± 2.5, 1.8 (0.6 to 3.5)
T2 White Matter Lesion Volume (mm3)		4636.9 ± 5775.2, 2589.4 (991.5 to 6133.2)
Normalized Thalamic Volume at Study Entry (thalamic volume/ICV*1000)		9.3 ± 1, 9.3 (8.7 to 9.9)

*CIS* clinically isolated syndrome, *DMT* disease-modifying therapy, *ICV* intracranial volume, *RRMS* relapsing remitting multiple sclerosis, *SPMS* secondary progressive multiple sclerosis, *T2* T2-weighted MRI.

*Mean ± SD, Median (Q1, Q3).

### Longitudinal normal aging data augmentation from large cross-sectional data

Multivariate Adaptive Regression Splines (MARS) was used to fit a cross-sectional spline so that we could augment the longitudinal data. MARS was chosen because of its robustness to outliers and its ability to auto-search non-linear associations with high dimensional interactions^41^. Age at scan, intracranial volume (ICV), and sex were used, with three-way interactions among them, as predictors of thalamic volume (percent of total brain volume). ICV and sex were treated as constant for each individual subject when augmenting the longitudinal data. Longitudinal thalamic volumes were augmented at ± 2 years from age at scan. We reserved 433 repeated measurements from 229 individuals as independent testing. ICC two-way mixed with absolute agreement and repeated measure correlation were used to assess the agreement/correlation between MARS model-augmented longitudinal data versus observed testing longitudinal data. SAS9.4 ADAPTIVEREG was used to fit the MARS model.

### Mixed spline model of thalamic atrophy trajectory

After data augmentation, we fit the mixed spline model. Let *n* be the number of subjects. For the *i*th participant, denote *t*_*i*_ as the age, denote $Y_{ij} (t)$ as the thalamus volume at the jth measurement for subject i, and denote X_ij_ as other predictors such as sex. To model the age effects accurately and efficiently, we use a semiparametric model of the form given below:

$${Y}_{ij}\left(t\right)={\mu }_{ij}\left(t\right)+{X}_{ij}\beta +{\upsilon }_{i}\left(t\right)+{\epsilon }_{ij}\left(t\right), i=1, \dots ,n, j=1, \dots , k$$

where ${\mu }_{ij}\left(t\right)$ is the unspecified aging trajectory for subject i at the jth time evaluated at age *t*, and *β* are the regression coefficients of the other predictors at the tj time. ${\upsilon }_{i}$ is the random effect of each subject. The measurement errors ϵ_*ij*_ are assumed to follow a normal distribution *N*(0,R), where R is the covariance matrix. This semiparametric regression model is a parsimonious way to both capture the potential nonlinear age trajectory and investigate the effects of other predictors. The simplest special case of this model is the linear mixed model where ${{\mu }_{i}}_{j}\left(t\right)$ = $\beta_{0i} + \beta_{1i} t_{ij}$. Regression splines are a broader class of models and could be fitted under this framework, which can be based on truncated power function (TPF) basis, B-spline basis or natural spline basis. These models vary by the choice of the spline basis and tuning parameters (the number of knots and the knot positions) that have an impact on the estimated shape of a spline function. Parameter-function estimation contains two major steps: (i) approximation using basis functions (e.g., TPF, B-Spline) which allows to fit lower-order polynomials within very small interval partitions (based on knots) and (ii) smoothing the approximation via penalty (e.g., random SPLINE coefficients, TOEPLIZ G-side matrix, RSMOOTH G-side matrix). The smoothing could be done via generalized cross-validation (GCV)^42^ or mixed effects approaches^43,44^, which are known to facilitate the choice of the knot positions in spline modelling^45^. They also allow a *penalty* to be applied directly to the model coefficients (P-spline penalty penalizes the squared differences between adjacent model coefficients, which in turn penalizes wiggles).

We then compared penalized splines (P-spline) with B-spline basis and truncated power function (TPF) basis with different random effect structures such as P-SPLINE and RSMOOTH (radial smoothing). For the P-spline, the unspecified function $\mu_{ij} (t)$ is approximated with a cubic B-Spline or TPF basis. Following Ruppert, Wand and Carroll^46^, the cubic spline can be represented as:

$${\mu }_{i}={\beta }_{0}+{\beta }_{1}{x}_{i}+{\beta }_{2}{{x}_{i}}^{2}+{\beta }_{3}{{x}_{i}}^{3}+{\sum }_{j=1}^{K}{\beta }_{3+j}({x}_{i}-{t}_{j}{)}^{3}$$

$$(x-t)=\left\{\begin{array}{c}x-t \, \, \, \, \, \, \, x>t\\ 0 \, \, \, \, \, \, \, \, \, \, \, \, {0}\end{array}\right.$$

The first part of the formula ${\mu }_{i}={\beta }_{0}+{\beta }_{1}{x}_{i}+{\beta }_{2}{{x}_{i}}^{2}+{\beta }_{3}{{x}_{i}}^{3}$ represents the polynomial function. The second part of the formula ${\sum }_{j=1}^{K}{\beta }_{3+j}({x}_{i}-{t}_{j}{)}^{3}$ represents the truncated power function (TPF). The ${t}_{j}$ represents the potential cut points for the knots. Estimation of parameters is made by minimizing the penalized log-likelihood function using proc GLIMMIX in SAS 9.4 with smoothing implemented using P-SPLINE smoothing (Random x/type = pspline) or radial smoothing (Random x/type = rsmooth). This mixed model formulation of spline smoothing has the advantage that the smoothing parameter is selected automatically^46^ and is shown to be more robust with misspecification of error dependence structure, compared to GCV-based approach^47^.

For the 12 spline structures described above, model comparison was made using four criteria: (i) Akaike information criterion (AIC) and Bayesian information criterion (BIC) criterion, with lower values indicating better fit; (ii) repeated measure correlation coefficient^48,49^ and intraclass correlation two-way mixed for longitudinal data between model-predicted versus observed data from reserved 10% testing dataset; (iii) visual inspection of the expected shape for projected lifespan spline (the normal curve must inherit the shape of the spline based on cross-sectional normal aging, and the MS curve must followed the shape of observed spaghetti plots as in Fig. 3A & Supplemental Fig. 10); and iv) both MS and normal aging trajectory curves must have narrow predicted interval along age points.

### Simulation study design

The purpose of the simulation study is to compare spline models to choose the most appropriate spline model for the “fish bone” data structure. The simulated data mimic the fish bone data structure by combining 10 sets of data from 10 different age blocks (*k* = 1 to 10) with age range from 30 to 80 by 5-year intervals (e.g., 30–34, 35–49). Each simulated data set was based on the covariance parameters estimated from a linear mixed model (with random intercept and slope). Block-specific weights (W_*k*_*,* V_*k*_) were added to the fixed effects of intercept and slope respectively for block *k*. W_*k*_ and V_*k*_ were altered to mimic a spline shape (“back bone” as shown in Fig. 1). The final mixed effects model was as follows:

$$ \begin{aligned} Y_{kij} = & W_{k} \beta_{k00} + V_{k} \beta_{k10} \left( {Year} \right)_{kij} + \beta_{k01} MS + \beta_{k11} \left( {MS} \right)*\left( {Year} \right)_{kij} + b_{k0j} + b_{k1j} \left( {Year} \right)_{kij} + \epsilon_{kij} , \\ \epsilon_{kij} \sim & iid N\left( {0, \sigma^{2} } \right) \\ \end{aligned} $$

The training sample was a combination of 10 datasets with 50 MS subjects each (age span 30 to 80 years). Each MS subject had 5 longitudinal MRI data points within each block, simulated using the linear mixed model above. Therefore, we simulated 500 subjects total in the training data. W_*k*_ and V_*k*_ started with small values in younger age, e.g. 1% decrease from the previous age block, but larger in middle age, e.g. 5% decrease, then became smaller again in older age, e.g. 1% decrease. The testing data followed the same simulation procedure, but we used the same subject ID across the 10 blocks; thus, the testing data contained 50 MS subjects, and each subject had 50 simulated age points. Because our ultimate goal is to predict the thalamic volume at an age that is younger than the observed age, the testing data included 4 more younger age points: 26, 27, 28, and 29, in addition to the 50 age points.

We considered twelve different models with three G-side covariance types (TOEPLIZ, P-SPLINE and radial smoothing) and four basis functions (Cubic-B-Spline, Cubic -TPF, Natural-TPF, Natural-B-Spline). To estimate the prediction accuracy from the spline model, we made comparisons using AIC/BIC with 500 iterations. The testing data were scored through each of the 12 spline models. We then obtained the estimated thalamic volume with associated 95% confidence interval at each age point. We took the average of the 500 replicates for the model-predicted thalamic volume and associated 95% CIs, then graphed the spline plots to visually inspect the overlap between true spline curve and the model-estimated spline curves, as well as the width of 95% confidence band.

### Real-life data application

We applied 12 different scenarios of spline models (listed in Table 3 to a real-life dataset with 519 MS subjects and 2053 normal aging subjects. For the normal subjects, we used augmented longitudinal data with 5 follow-up years (actual scan year at the middle). Among the 519 MS subjects, we randomly selected and reserved 52 MS subjects as the independent testing data. We repeated this iteration 10 times with 10 mutually exclusive independent testing data. For the first iteration, we selected the optimal spline structure and covariates using the criteria defined in 3.3. For covariate selection, we used a forward then backward strategy. Age spline, MS status, and age spline × MS status interaction were mandatory for each model. Other covariates included sex, baseline thalamic volume (Thalamus_0_), baseline ICV (ICV_0_), age of clinical onset (set as 0 for normal control), and cumulative years of exposure to MS disease modifying therapies at the first scan (DMT_0_; set as 0 for normal control). Each covariate entered the model first as the main effect, then as interaction terms with MS status or/and age spline. We categorized the covariate structure as the following: (a) only the main effect from each covariate; (b) two-way interactions with MS status, each covariate interaction term one by one, then multiple interaction terms together; (c) three-way interactions for each covariate one by one with MS status and age spline except DMT_0_ and age of clinical onset; (d) select any two covariates with the three-way interactions; (e) select any three covariates with the three-way interactions; (f) select any four covariates with the three-way interactions; (g) backwards selections with the terms showing model improvement from a to f. The same criteria defined in 3.3 were used for model selection. Once the final model was selected, we used the same model structure in the other 9 training datasets to obtain estimated coefficients, then applied them to independent testing data at each fold of iteration.

**Table 3:** AIC and BIC from different spline structures based on simulation data.

Cubic Spline Basis Function		G-side covariance type		
		TOEPLIZ	PSPLINE	RSMOOTH
Cubic-B-Spline	AIC	354.45 ± 74.55†	5843.49 ± 250.76	797.04 ± 80.81
Cubic –TPF	AIC	448.76 ± 117.7	5840.56 ± 250.33	809.23 ± 83.55
Natural-TPF	AIC	673.43 ± 76.93	5921.87 ± 250.77	906.61 ± 80.24
Natural-B-Spline	AIC	673.43 ± 76.93	5921.87 ± 250.77	906.61 ± 80.24
Cubic-B-Spline	BIC	421.88 ± 74.57	5910.93 ± 250.75	864.46 ± 80.76
Cubic–TPF	BIC	516.25 ± 117.71	5907.99 ± 250.33	876.67 ± 83.52
Natural-TPF	BIC	707.14 ± 76.9	5955.57 ± 250.75	940.34 ± 80.23
Natural-B-Spline	BIC	707.14 ± 76.9	5955.57 ± 250.75	940.34 ± 80.23

^†^Mean ± Std from 500 iterations.

For each independent testing MS patient, we constructed the hypothetical individualized normal aging trajectory curve (Health Digital Twin) and MS lifespan trajectory curve using the patient-specific covariates, which included sex, Thalamus_0_, ICV_0_, age of clinical onset (set as 0 for normal control), DMT_0_ (set as 0 for normal control) and sequential age points from 15 to 75 with an interval of 1. The age at which the MS trajectory curve began to depart from the HDT trajectory curve (or when both curves crossed in young age) was defined as the age of onset of progressive brain tissue loss. A bootstrapping procedure with 1000 iterations was used to determine the 95% confidence interval of this brain atrophy-defined age of onset. The bootstrapping procedure was conducted at the patient level, i.e., once a patient ID had been selected, all longitudinal scans associated with this patient were selected as a completed block. We repeated this procedure 10 times with 10 mutually exclusive independent validation data (tenfold cross validation procedure with 10% testing data each fold). Thus, each patient had an age of onset of progressive brain tissue loss (PBTL) with 95% CI estimated from their exclusive training dataset. In the end, we identified two groups of MS patients: earlier onset (the upper 95% CI limit of PBTL onset is younger than clinical onset age; in other words, the age of onset of PBTL was statistically significantly earlier than the age of clinical onset); and simultaneous onset (clinical onset did not differ statistically from the age onset of PBTL). We examined the different patterns of the onset age gap (age of clinical onset minus the age of onset of PBTL) between the earlier onset and simultaneous onset groups used Bland–Altman plots.

## Results

### Accuracy of augmented longitudinal normal aging data

**Figure 2:**
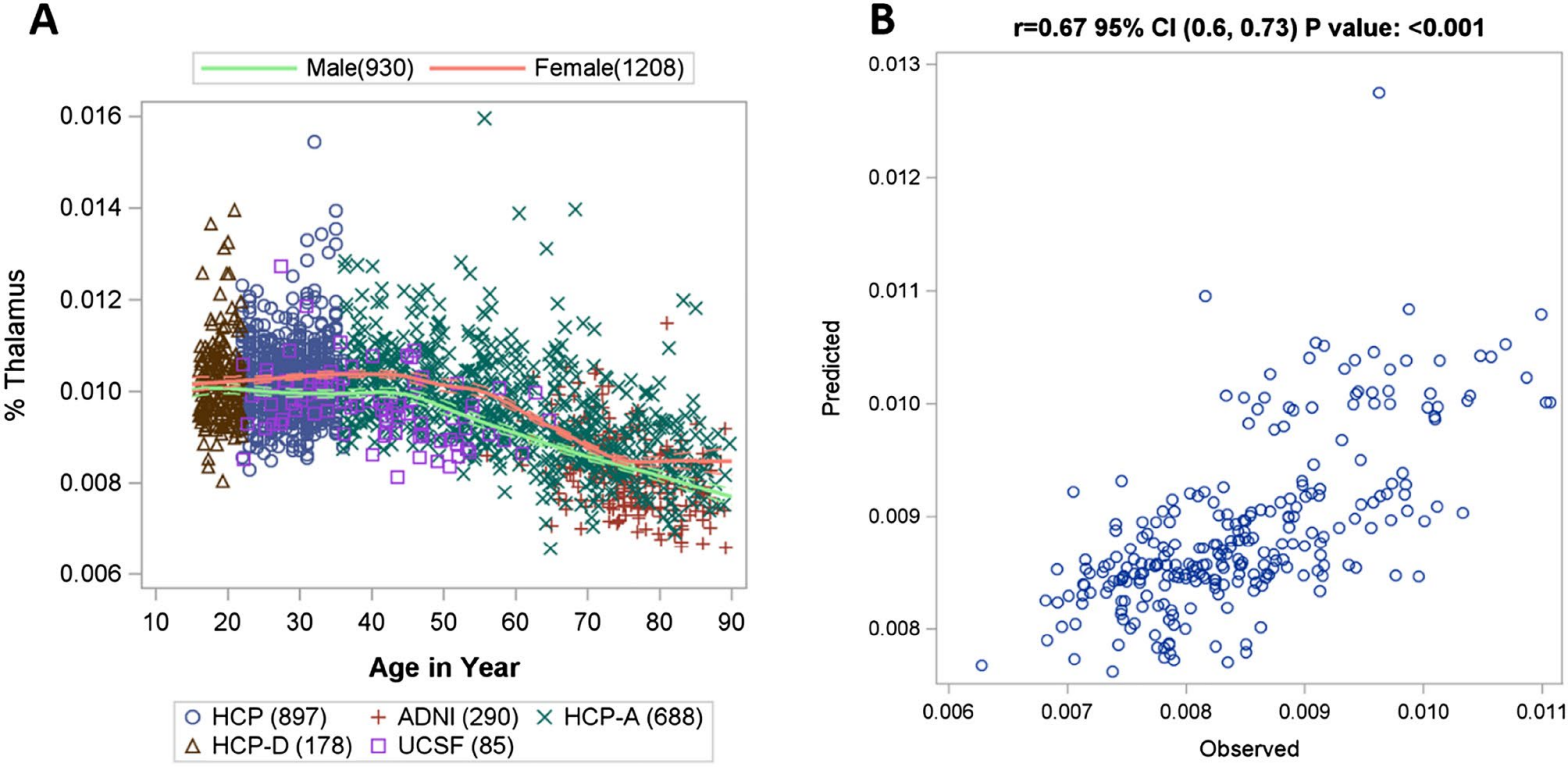
(**A**) Distribution of normalized thalamic volume (%Thalamus) in healthy controls across age from cross-sectional data (estimated using MARS). (**B**) Correlation between MARS model-predicted (i.e. augmented) and observed longitudinal data. A High-resolution version of this Figure can be found in Supplementary Information 2.

Figure 2A shows the smooth data cloud across age for healthy controls. The spline constructed by MARS represents the trend in the data. After data augmentation, independent validation was conducted by comparing the MARS model-predicted longitudinal data vs. the 433 observed longitudinal data. The model-predicted data had good agreement with the observed longitudinal data with ICC 0.62 95% CI (0.56, 0.68), using two-way mixed with absolute agreement. The predicted value explains 45% of total variance in the observed value (r = 0.67, 95% CI (0.6, 0.73), *p* < 0.001; R = 0.45) based on repeated measure correlation (Fig. 2B).

### Results of simulation study

**Figure 3:**
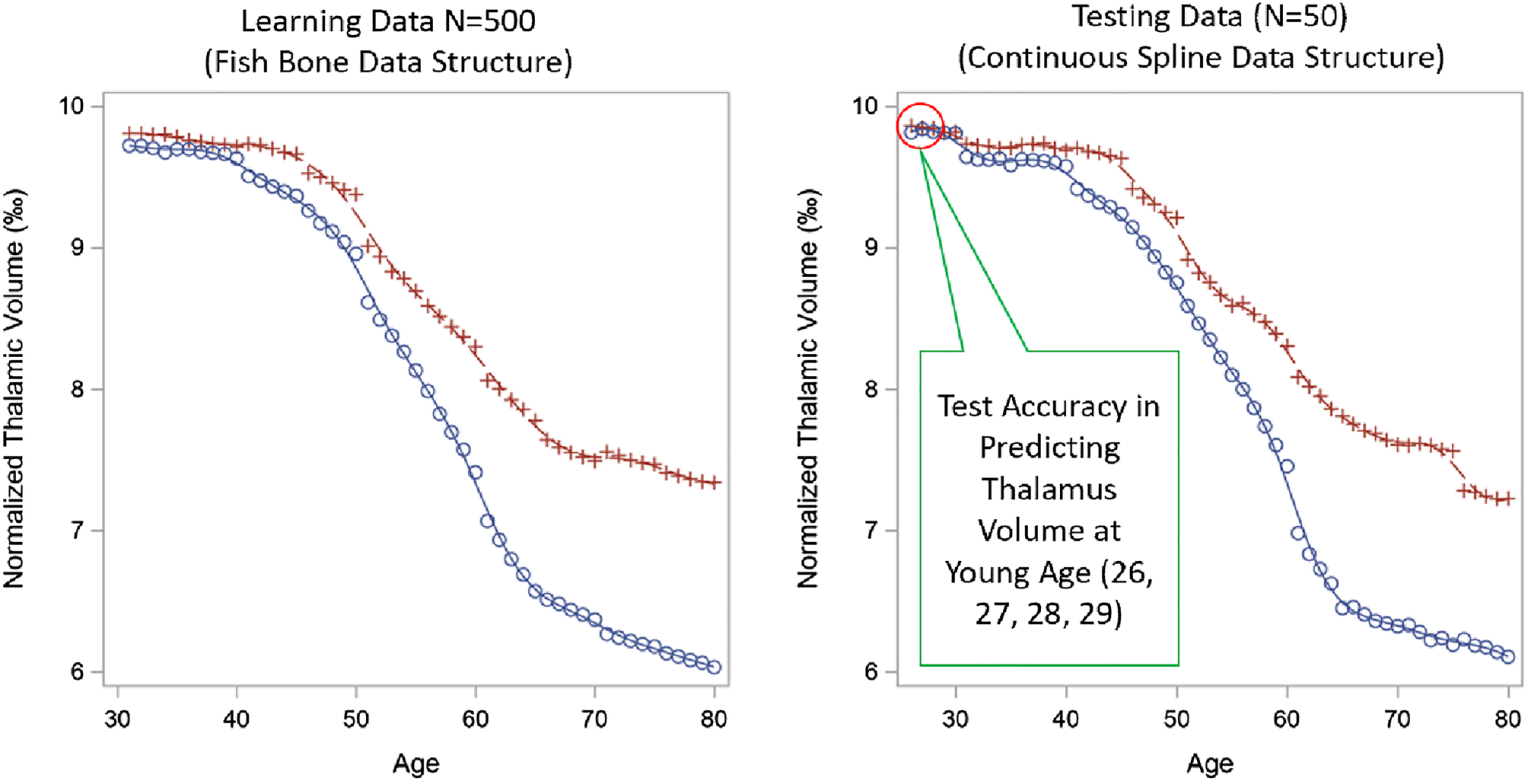
Simulated data curves for normal aging (red line) and MS (blue line). Description of simulated spline shape based on piecewise linear mixed models with age varying slopes. Red line represents normal aging and blue line represents MS. This illustrates the ‘ground truth’ spline shape that should be found by the mixed spline model. A High-resolution version of this Figure can be found in Supplementary Information 2.

Figure 3 shows the spline shapes for both simulated training and testing data from one of the 500 iterations, with red line as the normal aging trajectory and blue line as MS trajectory. The curve from the training data (left panel) shows the expected value of simulated ‘fish-bone’ structure which contains 10 datasets with 50 MS subjects each (age span 30–80 years). The curve from the testing data (right panel) shows the expected value of simulated ‘continuous spline’ data structure which contains 50 MS subjects with 50 simulated age points (referred as continuous age points across lifespan) plus 4 additional earlier age points (age 26–29). This represents the ‘ground truth’ spline shape that should be found by the mixed spline model.

Table 3 shows the mean and standard deviation of AIC and BIC comparing mixed-spline models and their corresponding G-side covariance from 500 iterations based on each of the 12 spline modeling scenarios. In general, unrestricted B-Spline had the smallest AIC or BIC showing the best fitting index, followed by unrestricted TPF. The restricted basis functions, both natural-B-Spline and natural-TPF, performed poorly. For G-side matrix, the TOEPLIZ had the best performance, followed by radial smoothing. P-SPLINE had the worst performance.

**Figure 4:**
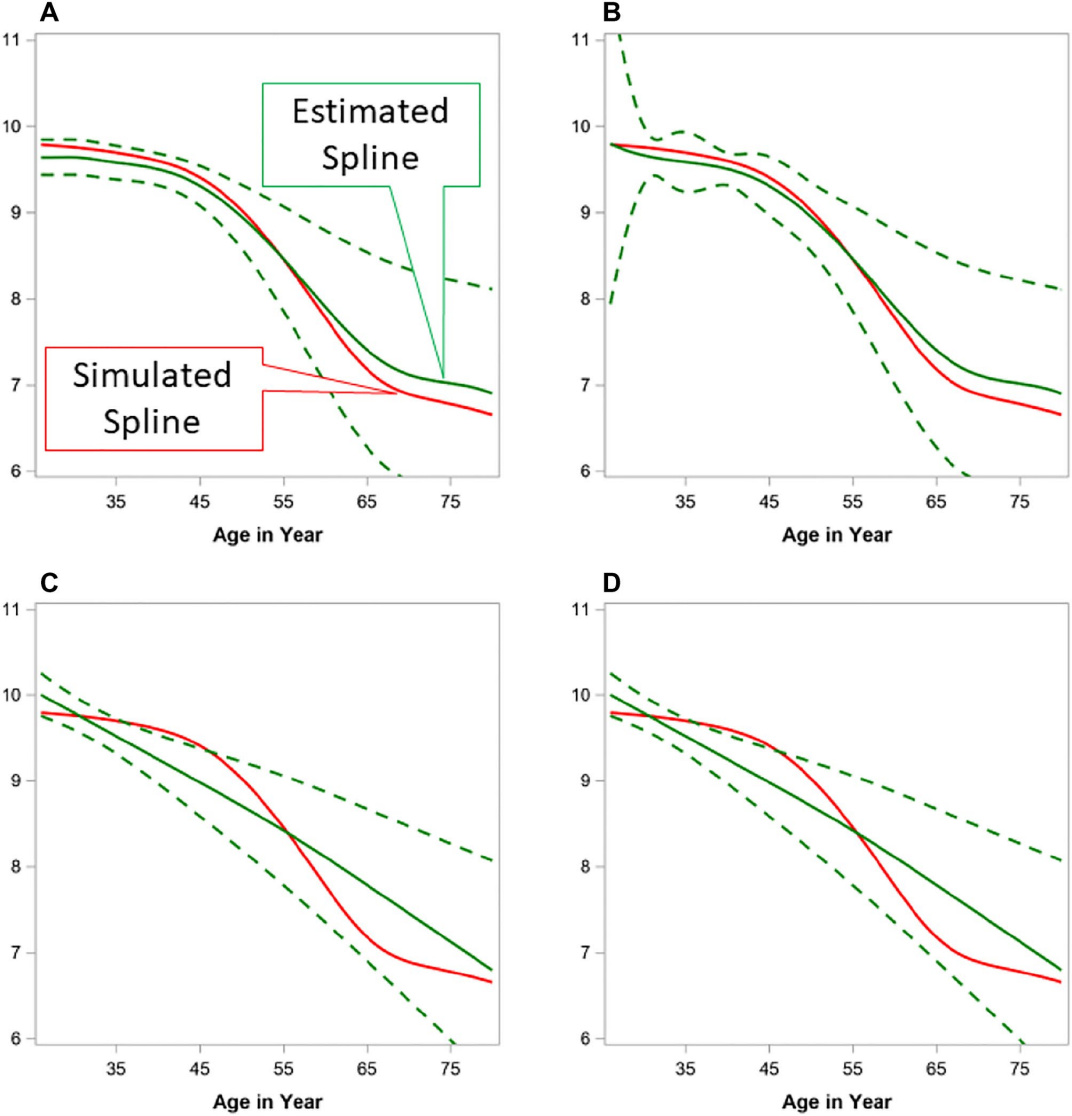
Illustration of simulated spline vs. predicted spline from simulation study. (**A**) cubic spline with bspline basis and random toep1; (**B**) cubic spline with TPF basis and random toep1; (**C)** restricted cubic spline with TPF basis and random toep1; (**D**) restricted cubic bspline with pspline basis and random toep1. A High-resolution version of this Figure can be found in Supplementary Information 2.

In addition to model fitting, we also assessed the prediction accuracy of each of the 12 spline models based on the prediction accuracy of the testing data. We visually inspected the overlap of the observed spline and the model-estimated spline curves, as well as the width of 95% confidence band. The visual inspection matched the AIC/BIC finding, with the best performance from B-Spline with a TOEPLIZ covariance model. The visual illustration of selected spline curves from TOEPLIZ is presented in Fig. 4.

Figure 4 shows the patterns of the smoothed spline from simulated value (ground truth) vs. the predicted value constructed using the mean of predicted values with 95% confidence band over 500 iterations from the testing data. In Fig. 4A, the predicted spline from TOEPLIZ with Cubic-B-Spline overlapped well with the ground truth spline, and the predicted 95% confidence band is narrow for the younger age. When using TPF as basis function (Fig. 4B), the 95% confidence band became very wide at early ages. The restricted splines (Fig. 4C,D) fitted a line as straight as a linear line, which is largely deviated from the simulated spline. Because our overall objective is to model the disease onset, we are most interested in modeling accuracy around the younger ages.

In summary, we have demonstrated through simulation that a mixed spline model can be used in a “fish bone” data structure when longitudinal MRI datasets only cover a few years of an individual’s lifespan. Figure 3 shows the ‘ground truth’ simulated spline shape based on piecewise linear mixed models with age varying slopes, and Fig. 4 shows good overlap between the ‘ground truth’ simulated spline and the estimated trajectory from the mixed spline model.

### Results of real-life data analysis

**Figure 5:**
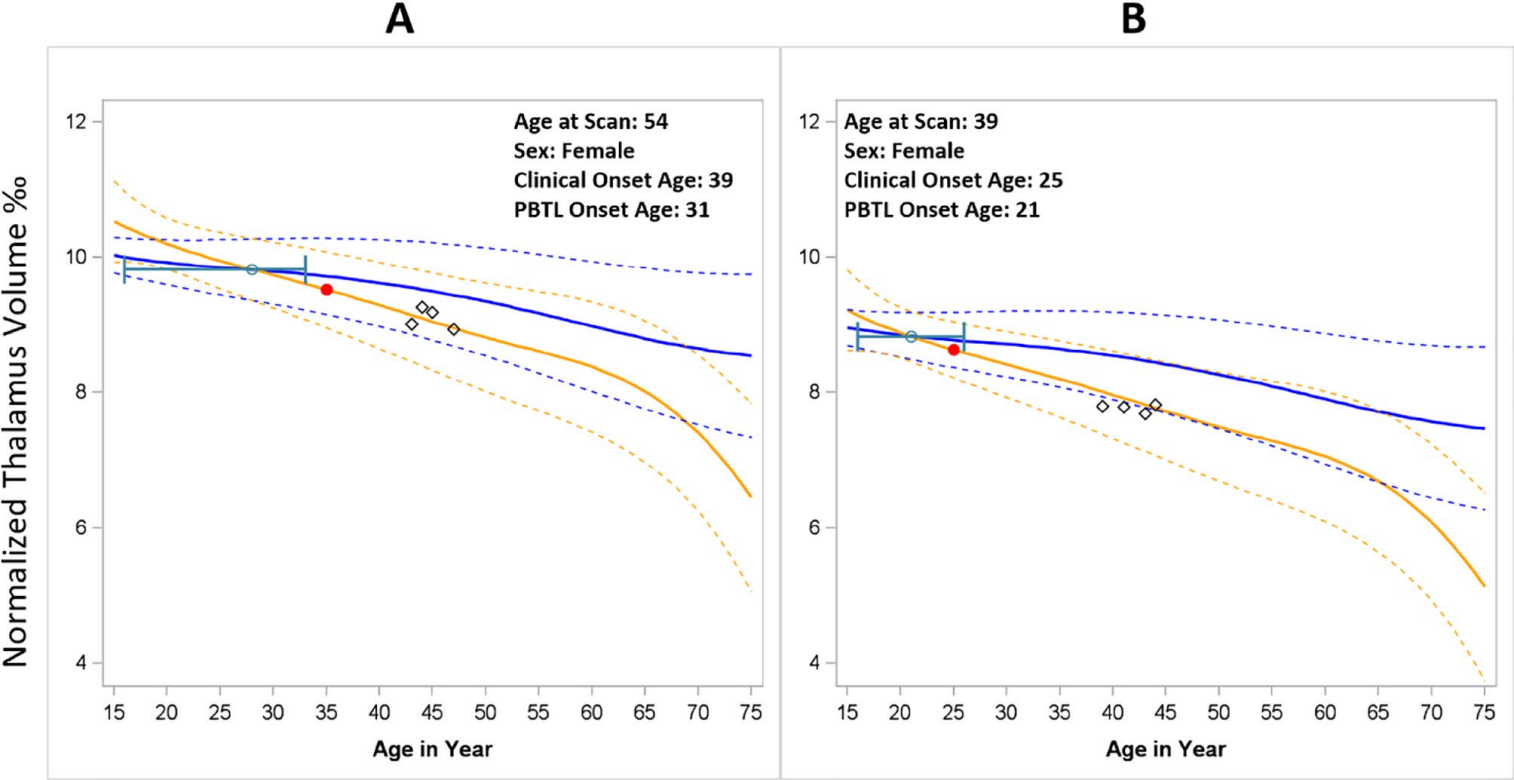
Trajectory curves for earlier onset (**A**) versus simultaneous onset (**B**). Estimation of Progressive Brain Tissue Loss (PBTL) in two individuals with MS (Panels A and B). The solid yellow line represents the individual MS subjects’ thalamic volume trajectories (95% CI = dashed yellow lines). The solid blue line represents the thalamic volume trajectory for a hypothetical normal aging individual with same demographic characteritics and baseline thalamic volume as the MS patient (their digital twin). 95% CI = dashed blue lines. The point at which the MS subjects’ trajectory deviates from their digital twin is defined as the onset of PBTL, denoted with the green dot (95% CI from bootstrapping in green). The red dot denotes the age of clinical onset. Black diamonds represent MS subjects’ observed thalamic volumes over time. A High-resolution version of this Figure can be found in Supplementary Information 2.

Supplemental Figs. 1–7 illustrate the model fitting indices of 12 spline structures and 52 covariate structures. As described in 2.3, the modeling fitting criteria included (i) AIC; (ii) repeated measurement correlation from 10% independent testing data between observed and predicted longitudinal values; (iii) shape of trajectory curves; and (iv) predicted 95% confidence band for trajectory curves. The real-life data application results concurred with the simulation study, with the best fitting model being the B-Spline with TOEPLIZ. The covariate structure included: age-spline, MS-status, ICV_0_, sex, sex*MS-status, Thalamus_0_, sex*age-spline, age at study entry, age of clinical onset, and DMT_0_. The final model reached a repeated measure correlation coefficient of 0.88 based on tenfold cross validation. Trajectory curves and scatter plots (Fig. 5, Supplemental Fig. 8) demonstrate that the spline curve ran through the observed data points in most of the cases (illustrated as black diamonds in Fig. 5). The AIC and BIC from the final model was also the smallest among all model structures.

Using this final model, we were able to construct the lifespan thalamic atrophy trajectory curve for an MS individual and their corresponding normal aging digital twin. Figure 5 illustrates two example cases. In each, the age of onset from progressive brain tissue loss (green dot) was younger than the age of clinical onset (red dot). The 95% confidence interval was derived from a bootstrapping procedure with 1000 iterations. Figure 5A shows that the upper limit of the 95% CI of the age of PBTL onset was younger than the age of clinical onset (earlier onset). In contrast, Fig. 5B shows that the 95% CI for the age of PBTL onset overlaps with the age of clinical onset (simultaneous onset).

**Figure 6:**
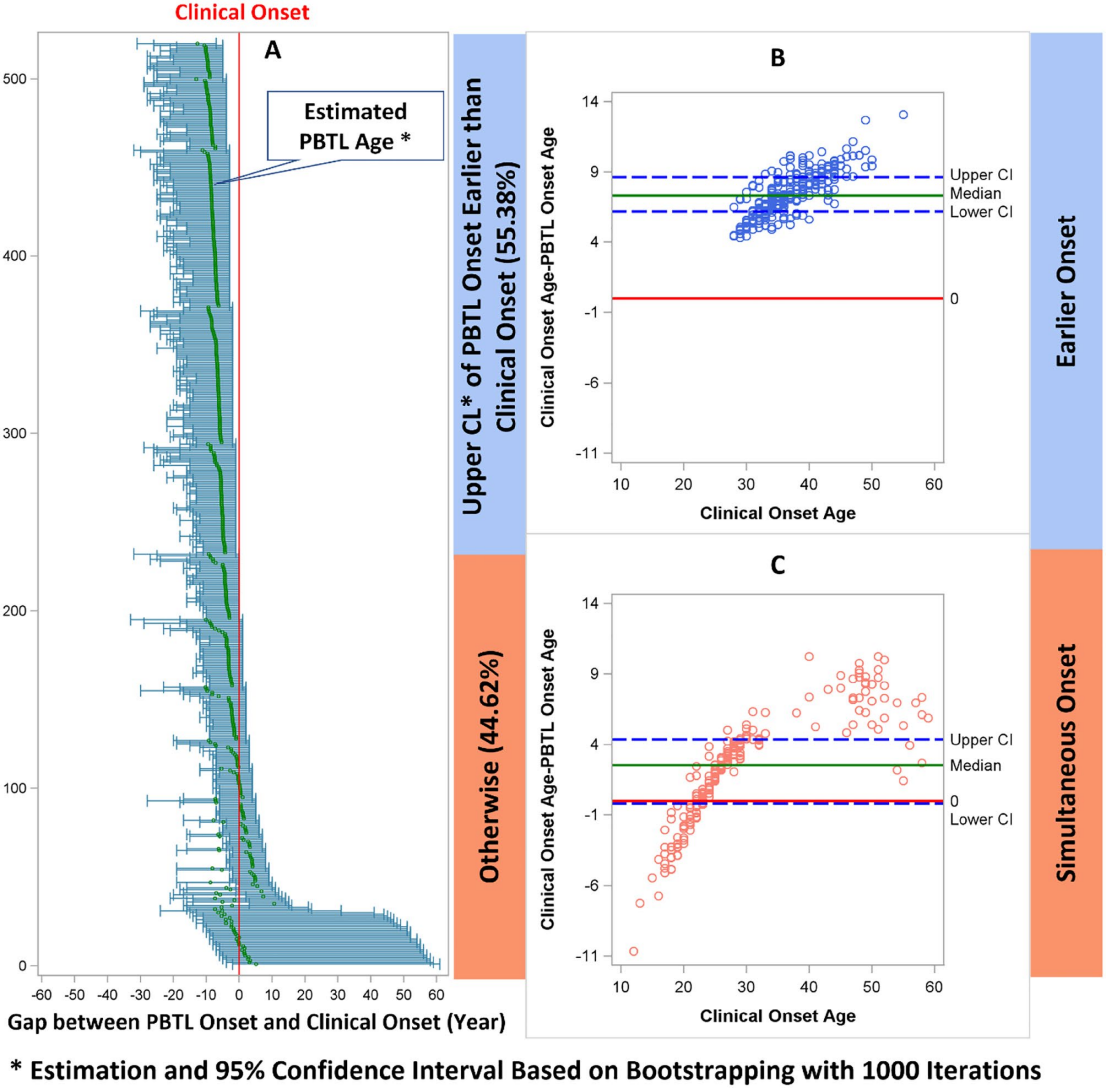
Panel A: Forest plot showing PBTL onset age minus clinical onset age (in years, x axis) for each of the 519 MS patients based on 1000 bootstrapping procedure. The center vertical red line represents the 0-reference line (clinical onset age). The tick marks in each horizontal line include the mean and 95% confidence interval. If the upper limit of 95% CI on the left of the center line, it suggests earlier onset. Otherwise, if the 95% CI includes the center line, it suggests simultaneous onset. Panels B and C: Bland–Altman plots. Each dot represents the difference between the age of onset of PBTL and the age of clinical onset. If the lower bound of 95% CI for the difference goes below the 0-reference line (red), it indicates weak evidence that the age of onset of progressive brain tissue loss is younger than the age of clinical onset. None of the dots are below the reference line in Panel B but they are in Panel C, consistent with our classification of “early” vs “simultaneous” onset. A High-resolution version of this Figure can be found in Supplementary Information 2.

Figure 6 shows the age of onset of progressive brain tissue loss based on 1000 bootstapping samples of 519 MS patients (Fig. 6A). The x-axis was centered by the age of clinical onset; it was calculated as age of PBTL onset minus age of clinical onset. Therefore, the center vertical line (red) is the zero reference line. If the upper limit of 95% CI is left of the center line, it suggests that the onset of PBTL was significantly earlier than the clinical onset (“earlier” onset). Otherwise, if the 95% CI includes the center line (0 gap between age of clinical onset and age of onset of progressive brain tissue loss), it suggests simultaneous onset. Overall, the age of onset of progressive brain tissue loss was younger than the age of clinical onset, with a mean difference of 5.1 ± 3.8 years and a median difference of 6 years (IQR 3.1–8.1).

Using our definitions, 55.4% of patients could be classified as earlier onset, while 44.6% of patient could be classified as simultaneous onset. In Fig. 6A, there are 26 patients with extremely high upper confidence limit of PBTL age. These patients (Supplemental Table 1) all had young clinical onset age and their mean projected PBTL onset age is close to the clinical onset age. Model fit was adequate in these patients and the wide upper confidence limits are mainly driven by outliers in the bootstrapping procedure. As such, they were correctly classified as “simultaneous onset.” Wilcoxon rank sum tests showed that earlier onset patients had statistically significantly older onset age compared to simultaneous onset, for both age of clinical (Median 36, IQR 33-41vs. Median 22, IQR 26–30, *p* < 0.01) and progressive brain tissue loss onset (Median 29, IQR 26–32 vs. Median 24, IQR 23–26, *p* < 0.01). Bland–Altman plots (Fig. 6B,C) showed age of onset of progressive brain tissue loss is much younger compared to age of clinical onset for patients in earlier onset group. Such difference is more scattered in the simultaneous onset group around the 0-reference line.

Demographics and disease characteristics between earlier and simultaneous onset patients are reported in Table 4. In general, patients with simultaneous onset were significantly younger at clinical disease onset and at study, were more likely to be male, had higher T2 lesion volumes, and had smaller thalamic volumes.

**Table 4:** Demographics and disease characteristics in patients with earlier onset versus simultaneous onset.

	Category	Earlier onset (n = 288)	Simultaneous onset (n = 231)	*p* value
Age at study entry (years)		44.5 ± 7.6, 43.9 (39 to 50)*	40.4 ± 11.6, 38.9 (30.7 to 50.8)	< 0.001‡
Sex	Female	226 (78.5%)	138 (59.7%)	< 0.001†
	Male	62 (21.5%)	93 (40.3%)	
Age of clinical onset (years)		36.9 ± 5.2, 36 (33 to 41)	29.3 ± 11, 26 (22 to 30)	< 0.001‡
disease type	CIS	59 (20.5%)	31 (13.4%)	0.11†
	RRMS	209 (72.6%)	182 (78.8%)	
	SPMS	20 (6.9%)	18 (7.8%)	
History of DMT use at study entry	Never used	125 (43.4%)	87 (37.7%)	0.19†
	Ever used	163 (56.6%)	144 (62.3%)	
DMT exposure (years)		n = 163, 2 ± 1.8, 1.6 (0.6 to 3.1)	n = 144, 3.1 ± 3, 2.1 (0.8 to 4.8)	0.013#
T2 white matter Lesion volume (mm3)		3776.4 ± 4916.8, 2106.3 (874.7 to 4546.2)	5719.3 ± 6553.2, 3456.5 (1191.1 to 7743.8)	< 0.001‡
Normalized thalamic volume at study entry (thalamic volume/ICV*1000)		9.4 ± 1, 9.4 (8.8 to 10)	9.1 ± 1, 9.2 (8.5 to 9.7)	0.012‡

*CIS* clinically isolated syndrome, *DMT* disease-modifying therapy, *ICV* intracranial volume, *RRMS* relapsing remitting multiple sclerosis, *SPMS* secondary progressive multiple sclerosis, *T2* T2-weighted MRI.

*Mean ± SD, Median (Q1, Q3), #Wilcoxon Rank Sum Test, ‡Independent T-Test, †Chi Square Test.

## Discussion

Health digital twin is a novel and promising concept to further advance precision medicine. It includes many major components such as personal devices, AI algorithms, right data to train the AI, and Internet of Things (IoT) for rapid data synchronization while providing real time decision-making^22^. The AI component can be considered the heart of the health digital twin approach. In aging-related fields such as neurodegenerative diseases, such AI algorithms must be developed from the scope of lifespan.

In this study, we apply the health digital twin conceptual framework to build an AI algorithm in addressing a fundamental clinical problem in multiple sclerosis, which is to identify the disease-related onset of brain atrophy. By estimating the deviation of the thalamic atrophy trajectory curve of an individual MS patient from their corresponding hypothetical normal aging health digital twin, our major finding is that progressive brain tissue loss precedes clinical disease onset in MS by a mean of 5.1 ± 3.8 years and a median of 6 years (IQR 3.1–8.1). Although the onset of progressive brain tissue loss measured by MRI is not synonymous with the true biological disease onset, our results suggest a major improvement in estimating MS disease duration compared to the standard practice of defining the disease onset as the time of first clinical symptom. This may have significant implications for MS clinicians, researchers, and patients, and could lead to a fundamental shift in our disease understanding and, one day, determining its cause.

Our novel approach in developing this AI algorithm towards health digital twin has several innovations. The first innovation is the development of a novel statistical application of mixed splines to overcome the challenge of lacking lifespan longitudinal data. Longitudinal studies usually have limited sample sizes and short follow-up periods. Generally, high-quality longitudinal MRI datasets, such as those that use the same pulse sequences and scanner, only contain 3–5 years of follow-up. To overcome this challenge, we describe the concept of the “fish bone” data structure. Structuring the data this way can have two benefits: (1) augment longitudinal data from large cross-sectional data; (2) augment individual lifespan trajectory based on small fragments of follow-up periods over a widespread age range.

To demonstrate these benefits, we successfully fit a spline model from a large cross-sectional dataset across a wide age range that reflects the non-linear trajectory of brain atrophy across the lifespan, and then used this to augment the longitudinal data with a good repeated measures correlation with the observed data (r = 0.67, *p* < 0.001). Moreover, rather than using follow-up time since study entry per the conventional approach, we used age at follow-up as the time variable for longitudinal data. This approach has been used in a recent study to predict mild cognitive impairment in Alzheimer’s disease based on the brain atrophy trajectory pattern by different periods^50^. However, the authors only explored a quadratic term as the non-linear effect. We have advanced the modeling strategy to a mixed spline model.

When fitting the longitudinal spline (mixed spline) model with an uneven time scale and short follow-up period, it was not trivial to determine the best fit spline structure from 12 different candidate spline structures. In our study, we used both simulation and real-life data to reach the conclusion that the best fitting model is B-Spline with TOEPLIZ as G-side matrix. This may be due to the simplicity of the mixed spline structure when applied to this special data structure. Determining the appropriate covariates was also not trivial, as those that interact with the spline slope will alter the slope, while non-interaction terms will affect the elevation of the spline and parallel distance between the MS curve and the normal aging trajectory curve. Interactions can be two-way, 3-way, or higher dimensional interactions. Given this, the covariate selection cannot follow the conventional forward, backward, or LASSO approach^51^. The interaction term must be intact with the marginal effect as a bundle. When adding higher dimensional interactions, each piece of lower-level interaction terms must be intact, too. Therefore, we used a manual forward then backward covariate selection strategy.

Given these complexities, determining the final model with the best fitting spline structure and covariates is challenging. The conventional approach is to use AIC and BIC; more stringent criteria require cross-validation. We initially used AIC/BIC plus a tenfold cross validation using the repeated measure correlation between model-predicted and observed values. However, we observed that this approach can be misleading. When constructing the 12 × 52 lifespan trajectory curves (Supplemental Figs. 1^1^–7) for a given individual, we observed scenarios where fitting indices were strong (small AIC/BIC, large r), but the trajectory curve was wild. For example, Supplemental Fig. 5A row E_01, column 3, a model with 3 three-way interaction terms, is likely overfit, and the spline shape did not inherit the shape we observed in large cross-sectional data (Fig. 3A). This phenomenon could be due to model tracing for few observed data points in the middle of the lifespan while sacrificing the fit of both far right (older) or far left (younger) ends. Since both AIC/BIC and repeated measure correlation were driven by the observed data, these fitting indices misled the lifespan trajectory. We added two additional criteria for model selection: (i) visual inspection of the shape of projected lifespan spline (normal aging curve must inherit the shape of the spline from large population based cross-sectional normal aging data and individual MS curve must follow the shape from group estimates and spaghetti plots); (ii) both MS and health digital twin trajectory curves must have narrow predicted bands along the age span. We were able to identify the optimal model based on this approach.

Our normal aging data were combined from four different studies. As such, one potential issue could be data heterogeneity due to slight differences in MRI protocols and scanner settings. A common practice in neuroimaging is to use a statistical model such as neuro-ComBat to harmonize the data before conducting further analysis^52^. Since each individual dataset of normal aging represents a subset of the age category, age must be added to neuro-ComBat as a covariate. Supplemental Fig. 9 shows the distribution of both the original (A) and ComBat harmonized (B) percent thalamic volumes along with age. The data distribution did not change from the original to ComBat harmonized thalamic volumes. In fact, the original data had a very smooth cloud across age. The robustness of our normalized thalamus volumes from different scanners and settings may be due to the use of relative values (thalamus as a percentage of ICV) instead of using absolute thalamic volumes. In a phantom study, we found that using relative values was robust to different scanner settings and protocols^53^. Forcing age as a covariate in neuro-ComBat removes the spline effect, which contradicts the non-linear brain trajectory reported from other major studies^32^. Therefore, we used and retained the original percent thalamic volumes throughout the study.

Although our overall model fit was adequate, we observed an interesting pattern in some patients with a young clinical onset age (Fig. 6, Supplemental Table 1). Within our dataset of 519 MS patients, 26 had extremely high upper confidence limit of their PBTL age. All of these patients had a young clinical onset age and a mean projected PBTL onset age that was close to their clinical onset age. Because they had young onset age, their trajectory curve will be close to and parallel with the normal aging trajectory curve. Thus, the age corresponding to the minimal distance between the two curves (when they cross) will be difficult to determine. In rare bootstrapping samples, the model identified an extreme older age as the PBTL onset age, and thus the wide upper confidence limits are mainly driven by outliers in the bootstrapping procedure. Those outliers will not affect our estimated PBTL onset age since we used the mean age from the bootstrapping sample, nor will they affect our classification of earlier vs. simultaneous onset. These patients very clearly belong to simultaneous onset group. However, this illustrates an issue that may arise in estimating PBTL age in patients with young clinical onset age and deserves future study.

Herein, we describe a novel statistical modeling strategy to overcome limitations in real-world longitudinal neuroimaging datasets and provide an application of the Health Digital Twin framework to address a fundamental clinical conundrum in MS. We found that the MS thalamic atrophy trajectory deviated from the corresponding hypothetical normal aging trajectory 5–6 years prior to clinical symptom onset. While there is no ground truth to validate our findings, this is consistent with clinical observations that white matter lesions Azevedoand thalamic atrophy are already present prior to first clinical symptoms in MS^28,30^. Further work should replicate the feasibility of using a mixed spline model for this type of data structure. We performed an initial comparison of disease characteristics and demographics in the two groups of MS patients we identified (early vs. simultaneous onset). While the focus of this paper is to describe the statistical methods, further investigations are underway to examine the clinical impact of these findings, which is outside the scope of the current work.

The notion that the biological disease onset may precede the first clinical symptoms is common across many neurologic and psychiatric diseases^54^. Because the trajectories of cortical, subcortical, grey matter, white matter, and whole brain volumes have been described over the lifespan^Bethlemen^, our method of leveraging these known trajectories can be applied to other neurodegenerative diseases according to their respective brain region of interest. Many neuropsychiatric disease states may benefit from this Digital Twin approach to accelerate the clinical use of precision medicine.

## Informed consent

Informed consent was obtained from all MS and single-site healthy control participants and was approved by the Committee on Human Research at UCSF. The local Institutional Review Board Committee at USC approved the use of additional anonymized brain MRI scans from healthy control participants. All methods were performed in accordance with the relevant guidelines and regulations.

### Supplementary Information

Supplementary Information.

Supplementary Figures.

## Data Availability

The datasets generated and/or analyzed during the current study are not publicly available due the condition and constraint from original sources. The data for the Human Connectome Project (HCP: http://www.humanconnectome.org), and Alzheimer’s Disease Neuroimaging Initiative (ADNI: http://www.adni-info.org) should be requested directly through the study website. The data from the single-center, prospective case–control cohort MS study was sponsored by Biogen and GSK. Authorization is needed for using the raw imaging data.
