# Author Correction: Toward precision medicine using a “digital twin” approach: modeling the onset of disease-specific brain atrophy in individuals with multiple sclerosis

**DOI:** 10.1038/s41598-023-47966-0

**Published:** 2023-12-15

**Authors:** Steven Cen, Mulugeta Gebregziabher, Saeed Moazami, Christina J. Azevedo, Daniel Pelletier

**Affiliations:** 1 Department of Radiology/Neurology, University of Southern California, Los Angeles, USA; 2 Department of Public Health Sciences, Medical University of South Carolina, Charleston, USA; 3 Department of Aerospace and Mechanical Engineering, Viterbi School of Engineering, University of Southern California, Los Angeles, USA; 4 Department of Neurology, Keck School of Medicine, University of Southern California, Los Angeles, USA

Correction to: *Scientific Reports* https://doi.org/10.1038/s41598-023-43618-5, published online 28 September 2023

In the original version of this Article, the Supplementary Information file which contains a high-resolution version of all the Figures was omitted.

The Supplementary Information file now accompanies the original Article.

