# Supplementary material for: Toward precision medicine using a “digital twin” approach: modeling the onset of disease-specific brain atrophy in individuals with multiple sclerosis
Source: Sci Rep. 2023 Sep 28;13:16279. doi: 10.1038/s41598-023-43618-5 (PMC10539386; doi:10.1038/s41598-023-43618-5)
Supplement: Supplementary file 1 — Supplementary Information. [file 41598_2023_43618_MOESM1_ESM.docx]

**Supplemental Figure 1: Model Fitting for Covariate Category A**


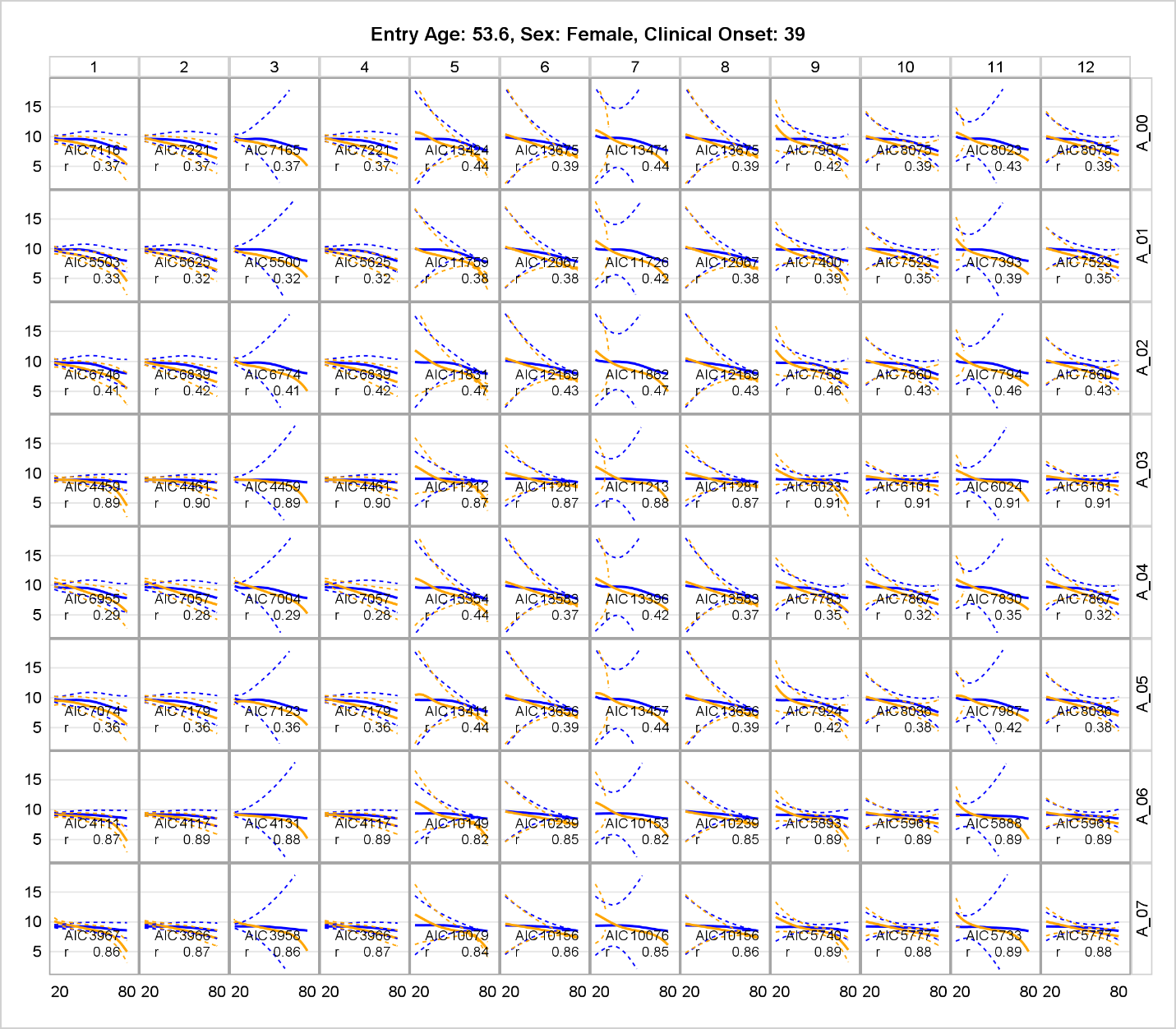


Column number 1-12 represents 12 different mixed spline structures:

1=cubic spline with bspline basis and random TOEPLIZ

2=cubic spline with tpf basis and random TOEPLIZ

3=restricted cubic spline with tpf basis and random TOEPLIZ

4=restricted cubic bspline with pspline basis and random TOEPLIZ

5=cubic spline with bspline basis and random pspline

6=cubic spline with tpf basisand and random pspline

7=restricted cubic spline with tpf basis and random pspline

8=restricted cubic bspline with pspline basis and random pspline

9=cubic spline with bspline basis and random radial smoother

10=cubic spline with tpf basisand and random radial smoother

11=restricted cubic spline with tpf basis and random radial smoother

12=restricted cubic bspline with pspline basis and random radial smoother

Row number A_00-A07 represents 7 models with different covariates (category A):

00: age-spline MS-status age-spline×MS-status

01: age-spline MS-status age-spline×MS-status ICV

02: age-spline MS-status age-spline×MS-status sex

03: age-spline MS-status age-spline×MS-status thalamus0

04: age-spline MS-status age-spline×MS-status age-of-onset

05: age-spline MS-status age-spline×MS-status DMT0

06: age-spline MS-status age-spline×MS-status ICV sex thalamus0 DMT0

07: age-spline MS-status age-spline×MS-status ICV sex thalamus0 DMT0 age-of-onset

**Supplemental Figure 2: Model Fitting for Covariate Category B**


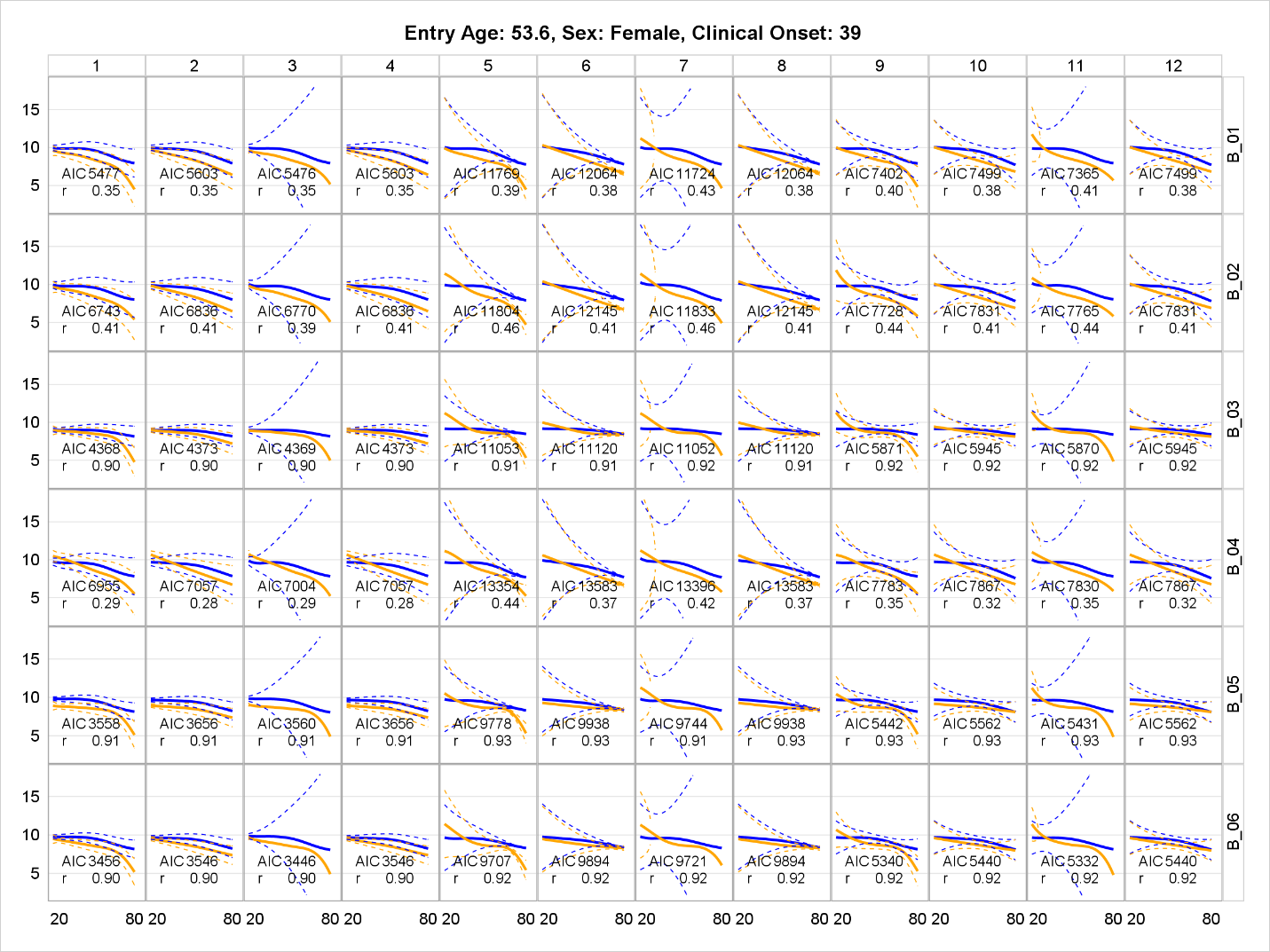


01: age-spline MS-status age-spline×MS-status ICV ICV×MS-status

02: age-spline MS-status age-spline×MS-status sex sex×MS-status

03: age-spline MS-status age-spline×MS-status thalamus0 thalamus0×MS-status

04: age-spline MS-status age-spline×MS-status age-of-onset

05: age-spline MS-status age-spline×MS-status DMT0 ICV ICV×MS-status sex×MS-status thalamus0 thalamus0×MS-status

06: age-spline MS-status age-spline×MS-status DMT0 ICV ICV×MS-status sex×MS-status thalamus0 thalamus0×MS-status age-of-onset

**Supplemental Figure 3: Model Fitting for Covariate Category C**


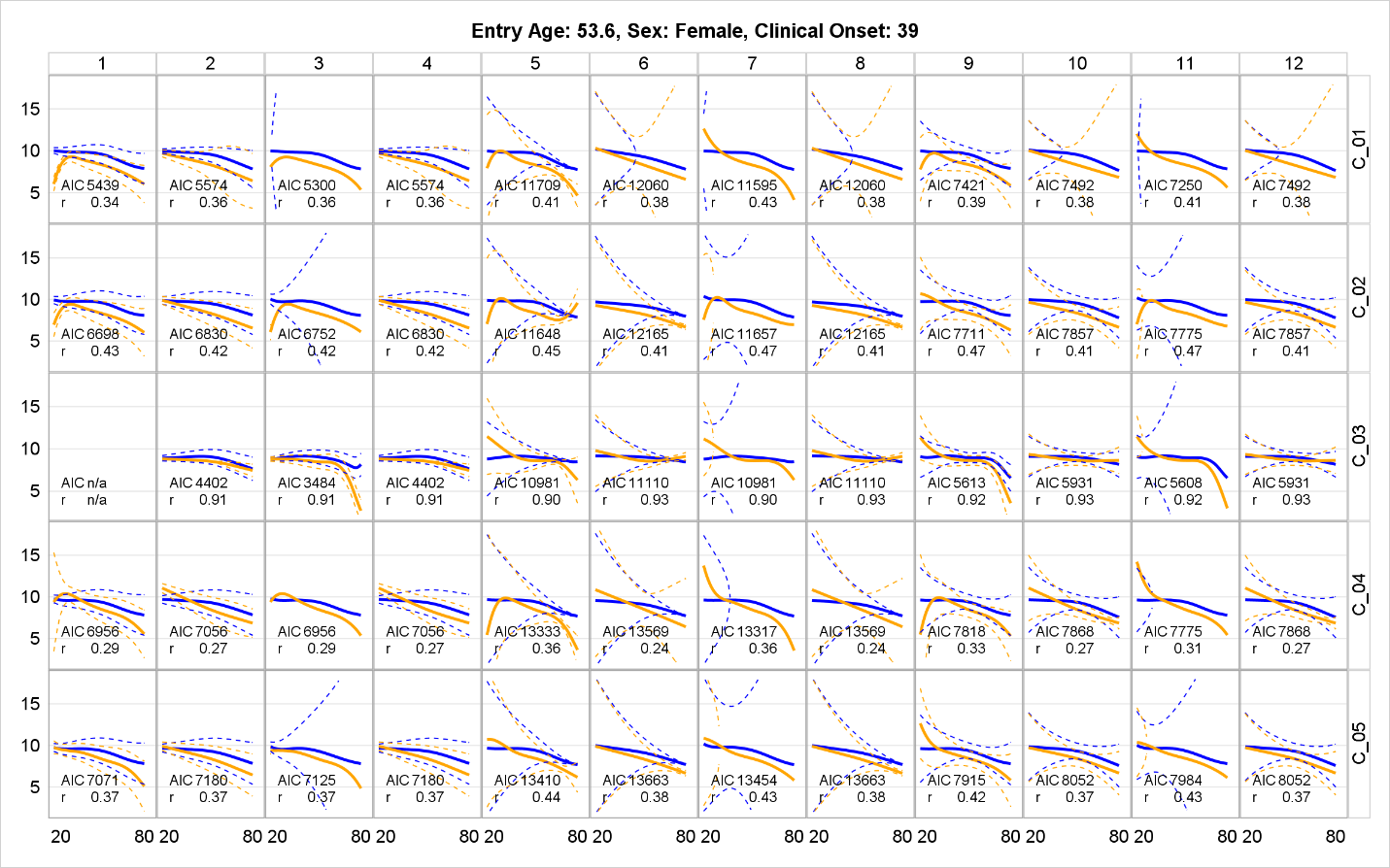


**X**: ICV ICV×MS-status ICV×age-spline ICV×age-spline×MS-status

**Y**: sex sex×MS-status sex×age-spline sex×age-spline×MS-status

**Z**: thalamus0 thalamus0×MS-status thalamus0×age-spline thalamus0×age-spline×MS-status

**V**: age-of-onset age-of-onset×age-spline

**W**: DMT0 DMT0×age-spline

01: age-spline MS-status **X**

02: age-spline MS-status **Y**

03: age-spline MS-status **Z**

04: age-spline MS-status **V**

05: age-spline MS-status **W**

**Supplemental Figure 4A: Model Fitting for Covariate Category D (01-05)**

**
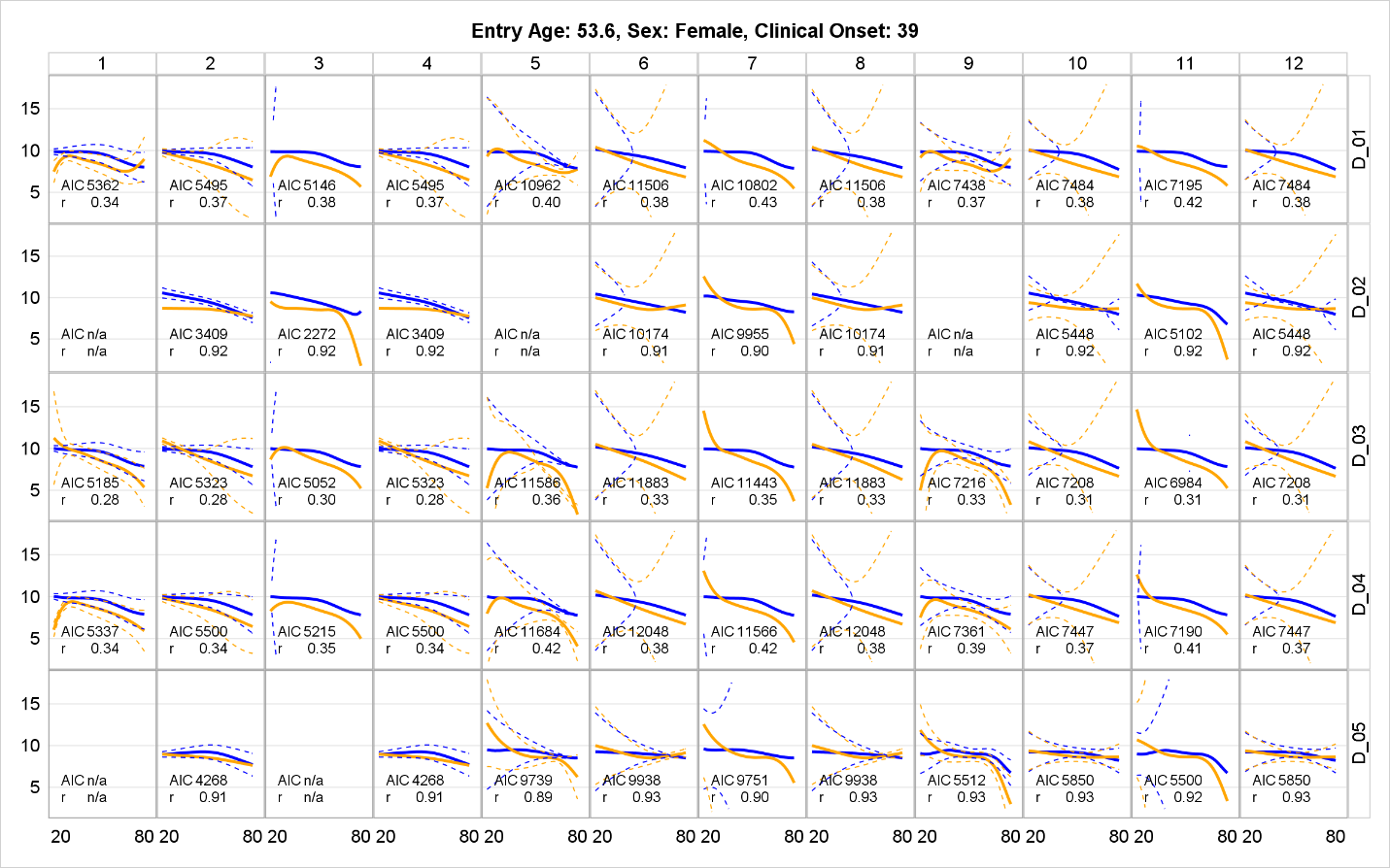
**

01: age-spline MS-status **X Y**

02: age-spline MS-status **Y Z**

03: age-spline MS-status **X V**

04: age-spline MS-status **X W**

05: age-spline MS-status **Y Z**

**Supplemental Figure 4B: Model Fitting for Covariate Category D (06-10)**


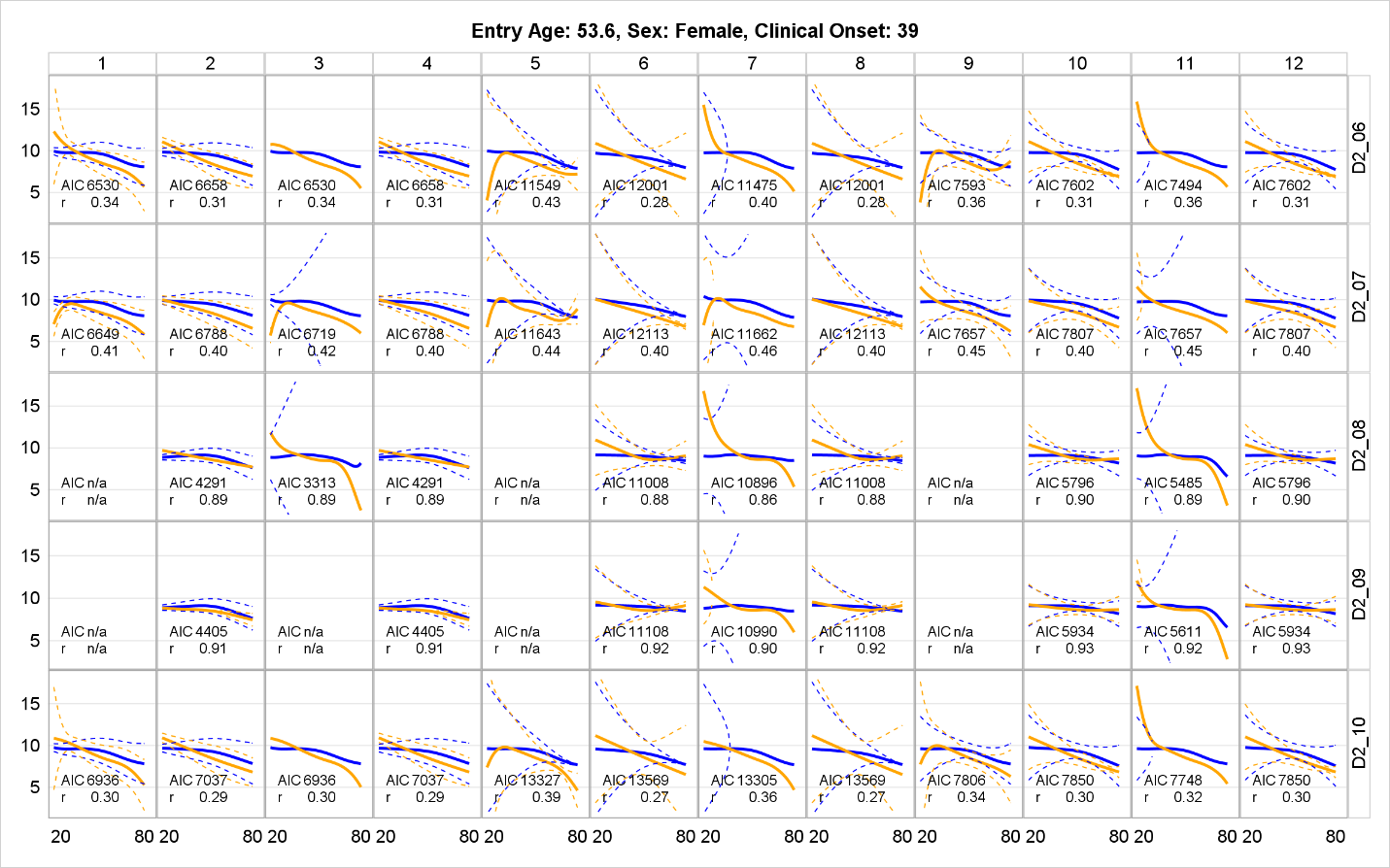


06: age-spline MS-status **X V**

07: age-spline MS-status **Y W**

08: age-spline MS-status **Z V**

09: age-spline MS-status **Z W**

10: age-spline MS-status **V W**

**Supplemental Figure 5A: Model Fitting for Covariate Category E (01-05)**

**
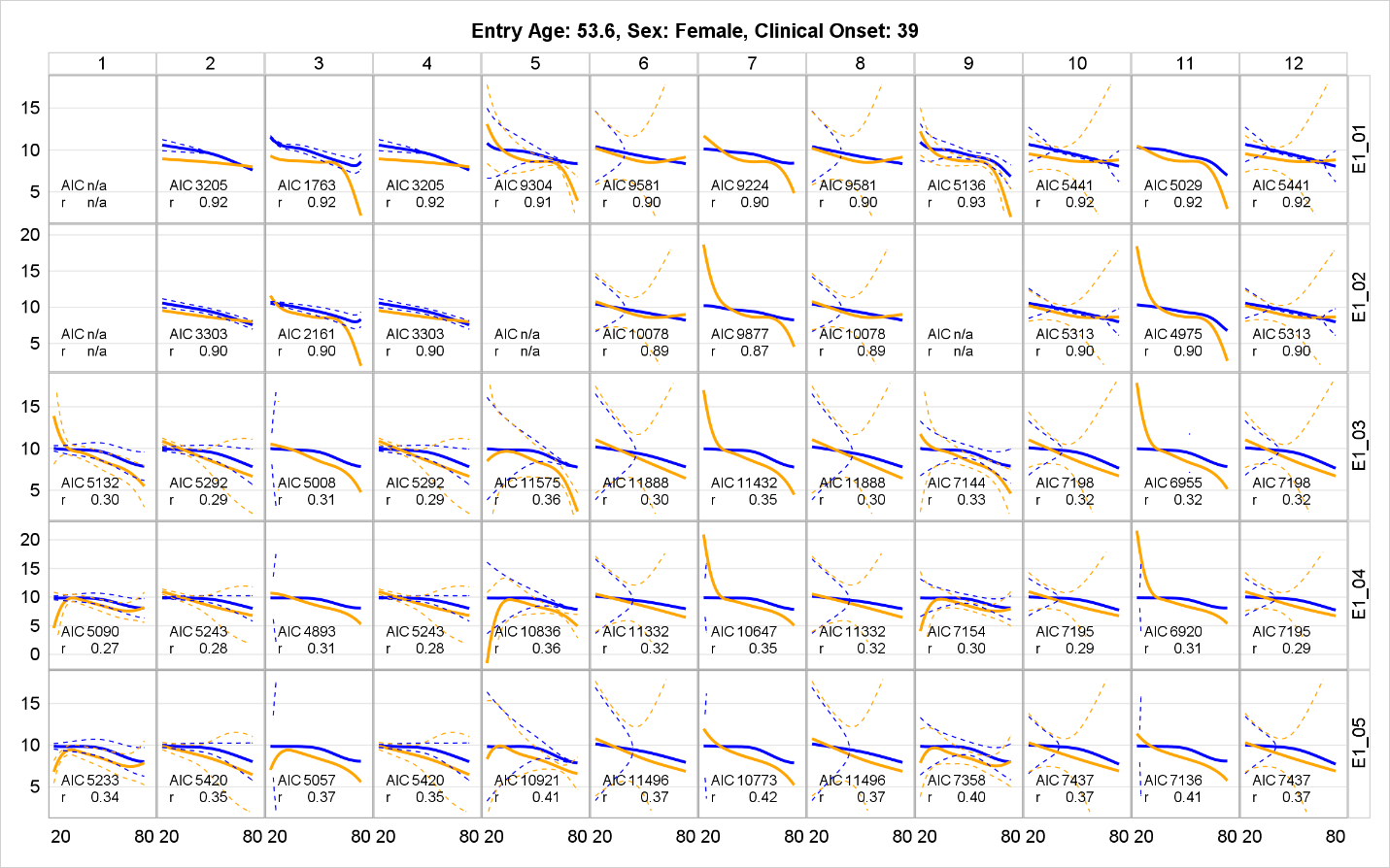
**

01: age-spline MS-status **X Y Z**

02: age-spline MS-status **X Z V**

03: age-spline MS-status **X V W**

04: age-spline MS-status **X Y V**

05: age-spline MS-status **X Y W**

**Supplemental Figure 5B: Model Fitting for Covariate Category E (06-10)**

**
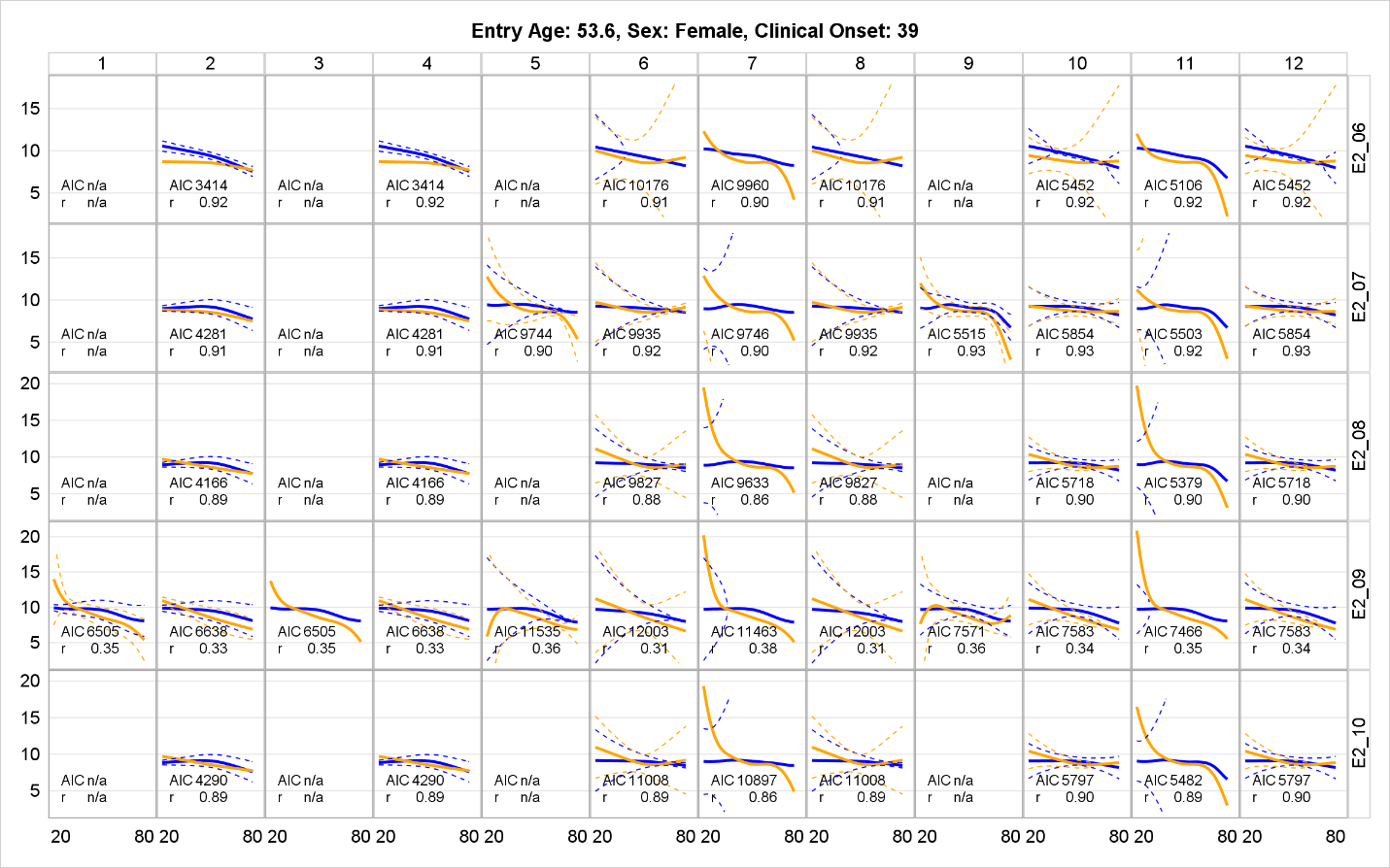
**

06: age-spline MS-status **X Z W**

07: age-spline MS-status **Y Z W**

08: age-spline MS-status **Y Z V**

09: age-spline MS-status **Y V W**

10: age-spline MS-status **Z V W**

**Supplemental Figure 6: Model Fitting for Covariate Category F (01-06)**

**
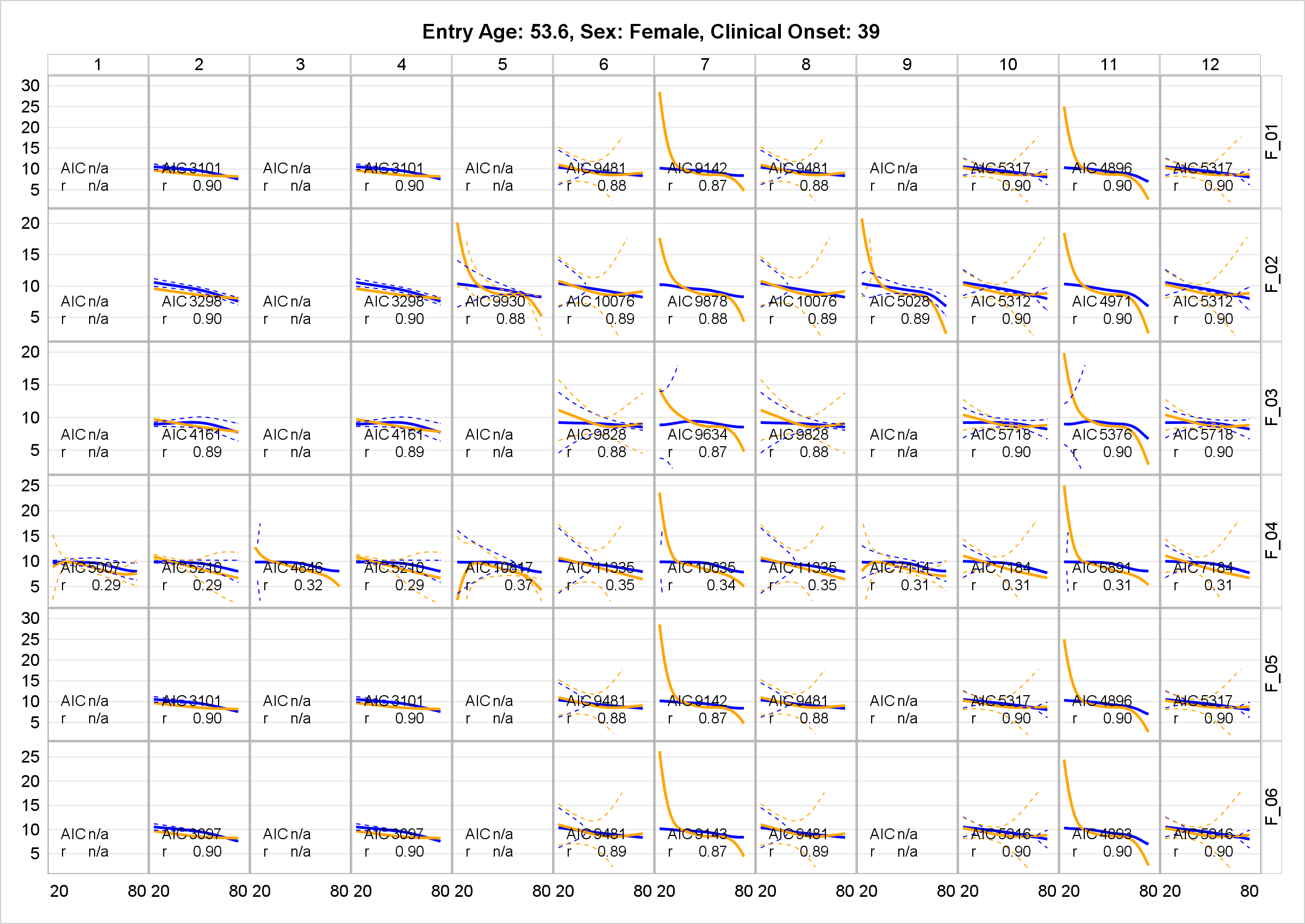
**

01: age-spline MS-status **X Y Z V**

02: age-spline MS-status **X Z V W**

03: age-spline MS-status **X Z V W**

04: age-spline MS-status **X Y V W**

05: age-spline MS-status **X Y Z V**

06: age-spline MS-status **X Y Z V W**

**Supplemental Figure 7: Model Fitting for Covariate Category G (01-06)**

**
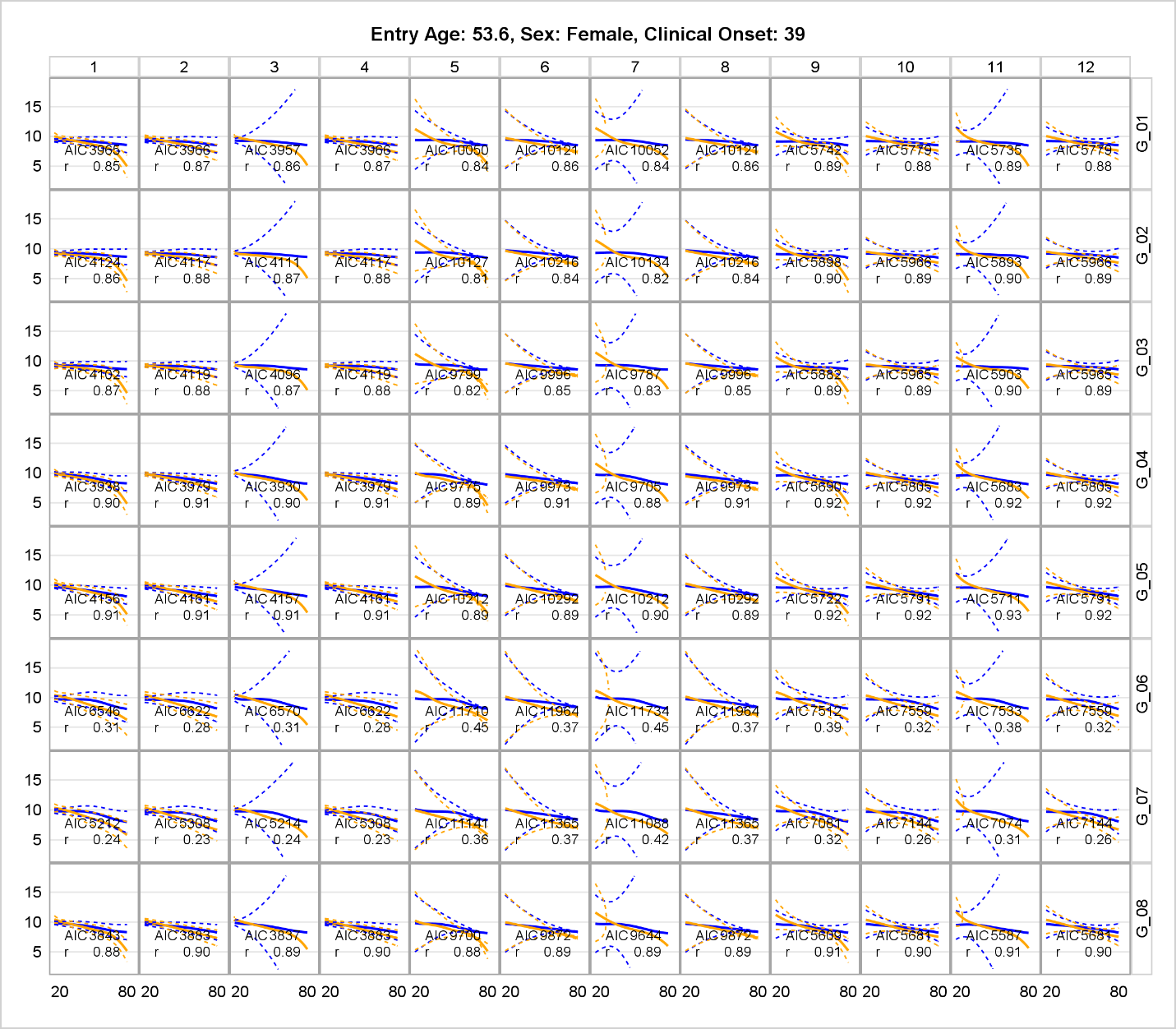
**

01: age-spline MS-status icv sex sex*MS-status Thalamus0 age-of-onset DMT0

02: age-spline MS-status icv sex sex*MS-status Thalamus0

03: age-spline MS-status icv sex sex*MS-status Thalamus0 sex*age-spline

04: age-spline MS-status icv sex sex*MS-status Thalamus0 sex*age-spline age-at-study-entry

05: age-spline MS-status sex sex*MS-status Thalamus0 sex*age-spline age-at-study-entry

06: age-spline MS-status sex sex*MS-status sex*age-spline age-at-study-entry

07: age-spline MS-status icv sex sex*MS-status age-at-study-entry age-of-onset DMT0

08: age-spline MS-status icv sex sex*MS-status Thalamus0 sex*age-spline age-at-study-entry age-of-onset DMT0

**Supplemental Figure 8: HDT and MS Trajectory Curves for 50 Individuals from Cross-Validation
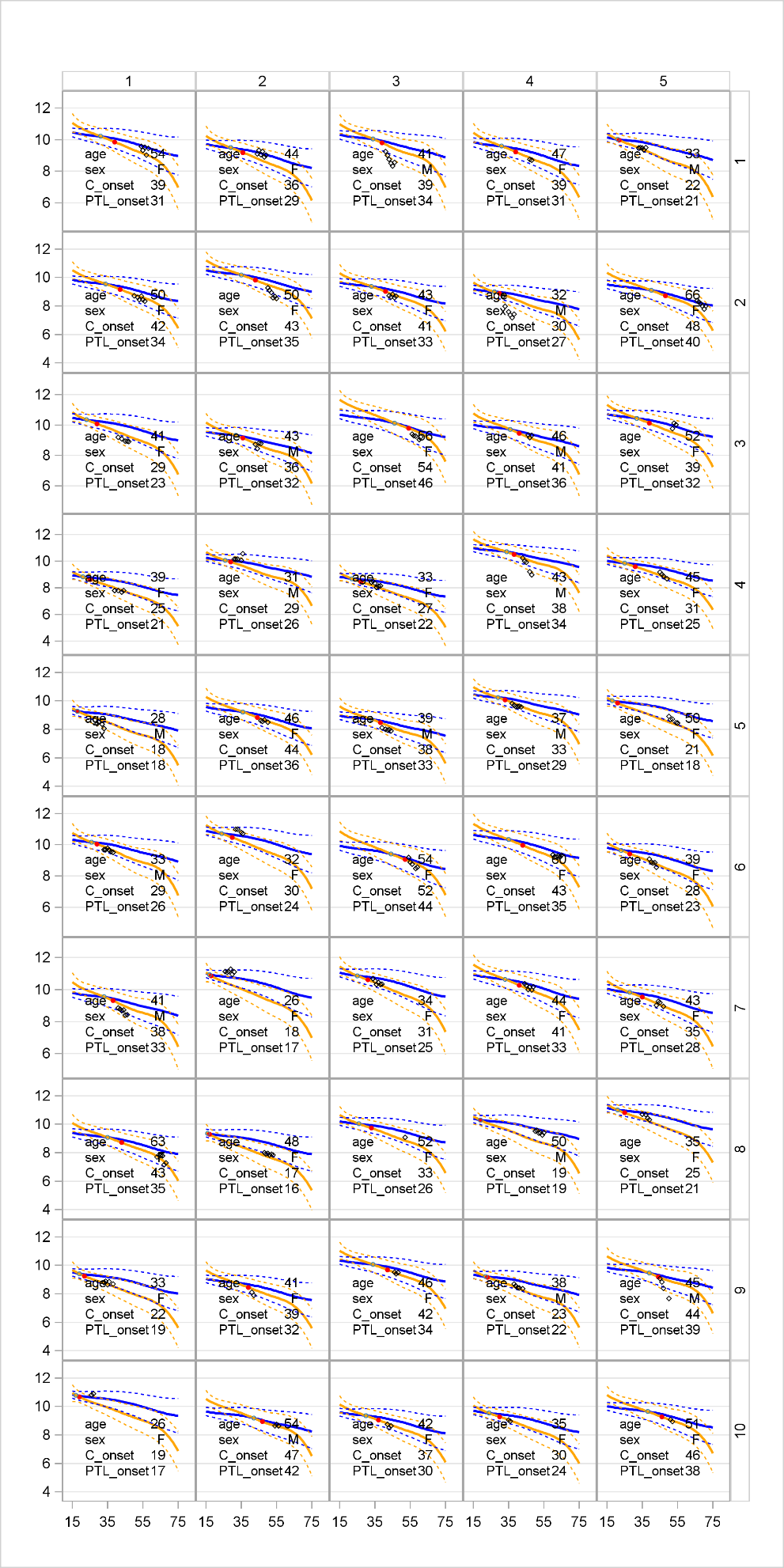
**

Rad Dots: Age of Clinical Onset (C_onset)

Green Circle: Age of Onset from Progressive Brain Tissue Loss (PTL_onset)

Black Diamond: Observed % Thalamus

**Supplemental Figure 9: Distribution of Percent Thalamus Volume before and after Neuro-Combat Harmonization**

**
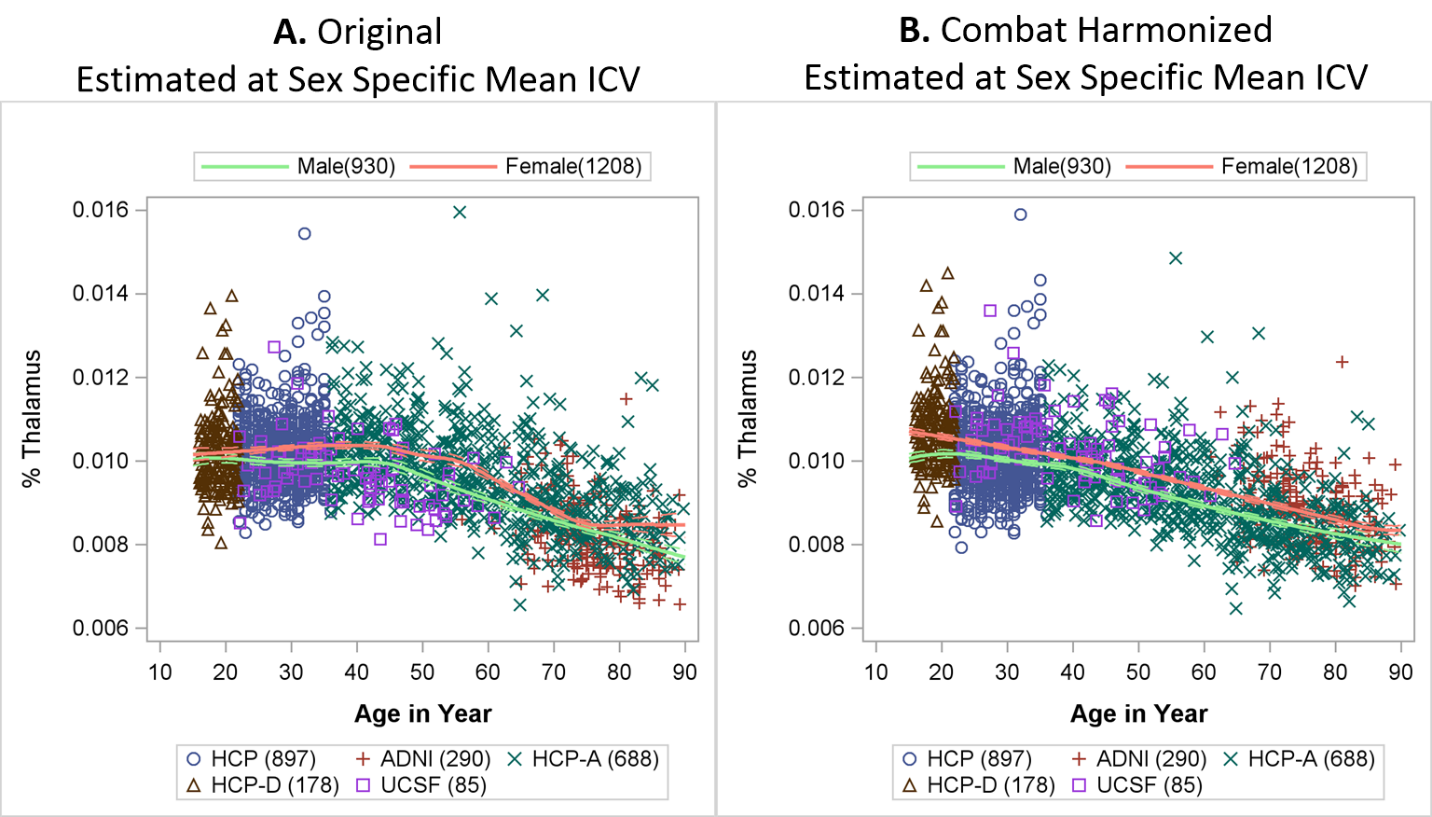
**

**Supplemental Figure 10: Spaghetti Plot for MS and Spline Trend Based on Baseline Data**


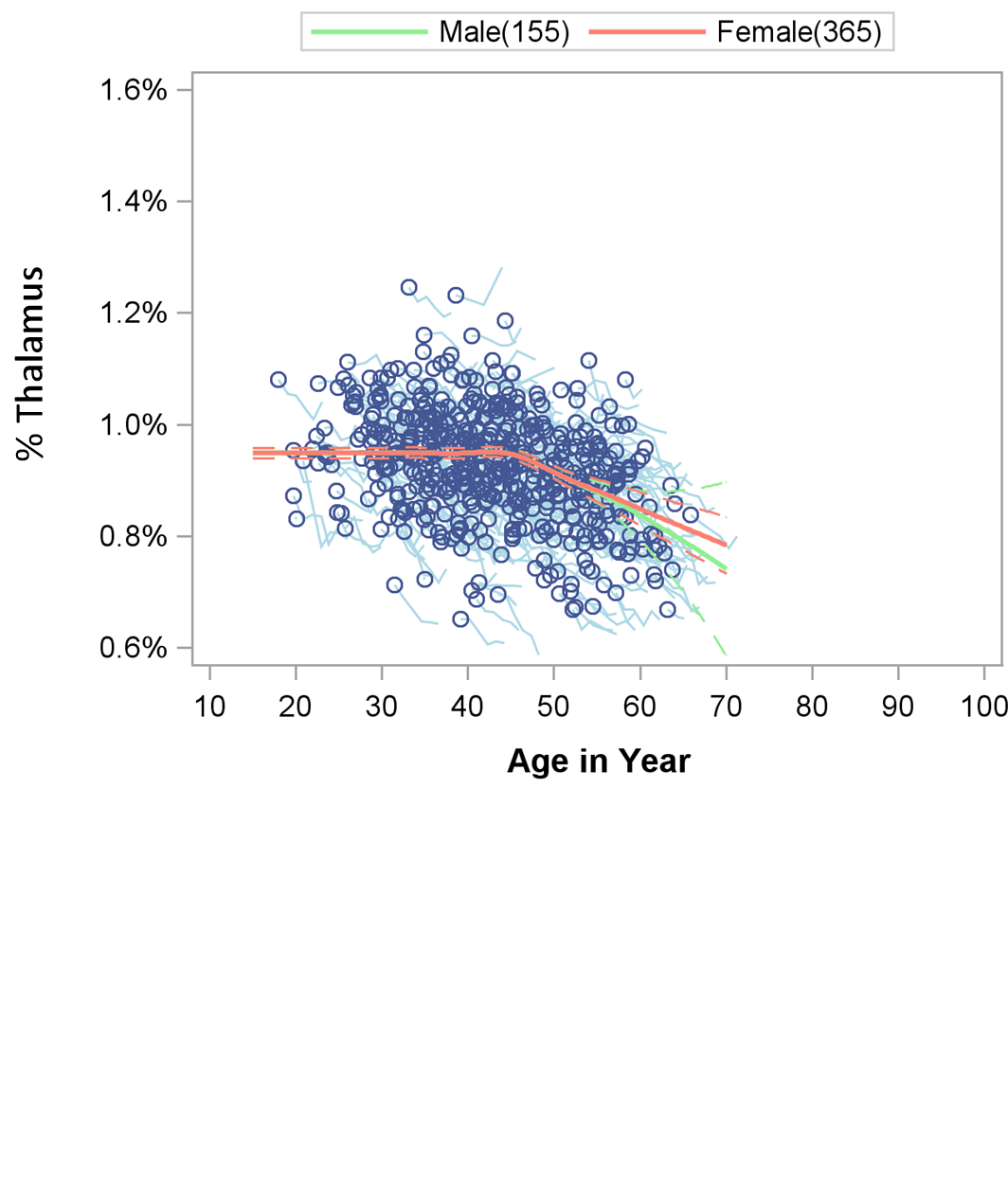


**Supplemental Table1: List of Patients with Extreme Upper 95% CI in Figure 6A**

| id | mean | std | Percentile 2.5 | Percentile 25 | Percentile 50 | Percentile 75 | Percentile 90 | Percentile 97.5 | low limit 95% CI | upper limit 95% CI | low limit centered | upper limit centered | Age of Onset |
| --- | --- | --- | --- | --- | --- | --- | --- | --- | --- | --- | --- | --- | --- |
| ms0405 | 23 | 10 | 15 | 20 | 22 | 23 | 25 | 76 | 15 | 42.5 | -9 | 18.5 | 24 |
| ms0513 | 22.8 | 12.1 | 15 | 18 | 21 | 23 | 25 | 77 | 15 | 46.4 | -5 | 26.4 | 20 |
| ms0522 | 22.5 | 10.3 | 15 | 19 | 21 | 23 | 25 | 76 | 15 | 42.8 | -6 | 21.8 | 21 |
| ms0528 | 23.5 | 10.9 | 15 | 20 | 22 | 24 | 25 | 77 | 15 | 44.8 | -11 | 18.8 | 26 |
| ms0532 | 23.3 | 9.7 | 15 | 20 | 22 | 24 | 25 | 76 | 15 | 42.3 | -10 | 17.3 | 25 |
| ms0583 | 22.7 | 11.4 | 15 | 19 | 21 | 23 | 25 | 77 | 15 | 45 | -8 | 22 | 23 |
| ms0686 | 23.2 | 12.3 | 15 | 19 | 21 | 23 | 25 | 78 | 15 | 47.2 | -9 | 23.2 | 24 |
| ms0713 | 23.6 | 11.9 | 15 | 20 | 22 | 23 | 25 | 77 | 15 | 47 | -10 | 22 | 25 |
| ms0732 | 22.1 | 11.9 | 15 | 17 | 20 | 23 | 25 | 78 | 15 | 45.4 | -2 | 28.4 | 17 |
| ms0737 | 22.5 | 11.2 | 15 | 19 | 21 | 23 | 25 | 77 | 15 | 44.4 | -6 | 23.4 | 21 |
| ms0759 | 23.6 | 9.4 | 15 | 21 | 22 | 24 | 25 | 75 | 15 | 42.1 | -11 | 16.1 | 26 |
| ms0811 | 22.9 | 11.7 | 15 | 19 | 21 | 23 | 25 | 77 | 15 | 45.8 | -8 | 22.8 | 23 |
| ms0823 | 23.5 | 10.6 | 15 | 20 | 22 | 24 | 25 | 76 | 15 | 44.2 | -9 | 20.2 | 24 |
| ms0836 | 22.1 | 12 | 15 | 17 | 20 | 23 | 25 | 78 | 15 | 45.6 | -4 | 26.6 | 19 |
| ms0843 | 22.9 | 9.8 | 15 | 20 | 22 | 24 | 25 | 75 | 15 | 42.1 | -8 | 19.1 | 23 |
| ms0885 | 23.2 | 11.6 | 15 | 19 | 21 | 23 | 25 | 77 | 15 | 45.9 | -7 | 23.9 | 22 |
| ms0905 | 24 | 10.4 | 15 | 21 | 22 | 24 | 26 | 76 | 15 | 44.4 | -13 | 16.4 | 28 |
| ms1080 | 22.3 | 11.2 | 15 | 18 | 20 | 23 | 25 | 77 | 15 | 44.3 | -4 | 25.3 | 19 |
| ms1096 | 24.1 | 9.3 | 16 | 21 | 23 | 25 | 26 | 73 | 16 | 42.3 | -11 | 15.3 | 27 |
| ms1113 | 22.6 | 12.8 | 15 | 17 | 20 | 23 | 25 | 78 | 15 | 47.6 | -5 | 27.6 | 20 |
| ms1131 | 22.7 | 12.4 | 15 | 18 | 20 | 23 | 25 | 78 | 15 | 47.1 | -6 | 26.1 | 21 |
| ms1162 | 23.6 | 9.2 | 15 | 21 | 23 | 24 | 25 | 73 | 15 | 41.7 | -11 | 15.7 | 26 |
| ms1180 | 22.5 | 11.2 | 15 | 19 | 21 | 23 | 25 | 77 | 15 | 44.4 | -6 | 23.4 | 21 |
| ms1192 | 22.9 | 12.2 | 15 | 19 | 21 | 23 | 25 | 78 | 15 | 46.8 | -8 | 23.8 | 23 |
| ms1495 | 22.9 | 12.7 | 15 | 18 | 21 | 23 | 25 | 78 | 15 | 47.8 | -7 | 25.8 | 22 |
| ms1543 | 23.4 | 9.9 | 15 | 20 | 22 | 24 | 25 | 76 | 15 | 42.7 | -10 | 17.7 | 25 |
