# Supplementary figures and images for: Toward precision medicine using a “digital twin” approach: modeling the onset of disease-specific brain atrophy in individuals with multiple sclerosis
Source: Sci Rep. 2023 Sep 28;13:16279. doi: 10.1038/s41598-023-43618-5 (PMC10539386; doi:10.1038/s41598-023-43618-5)

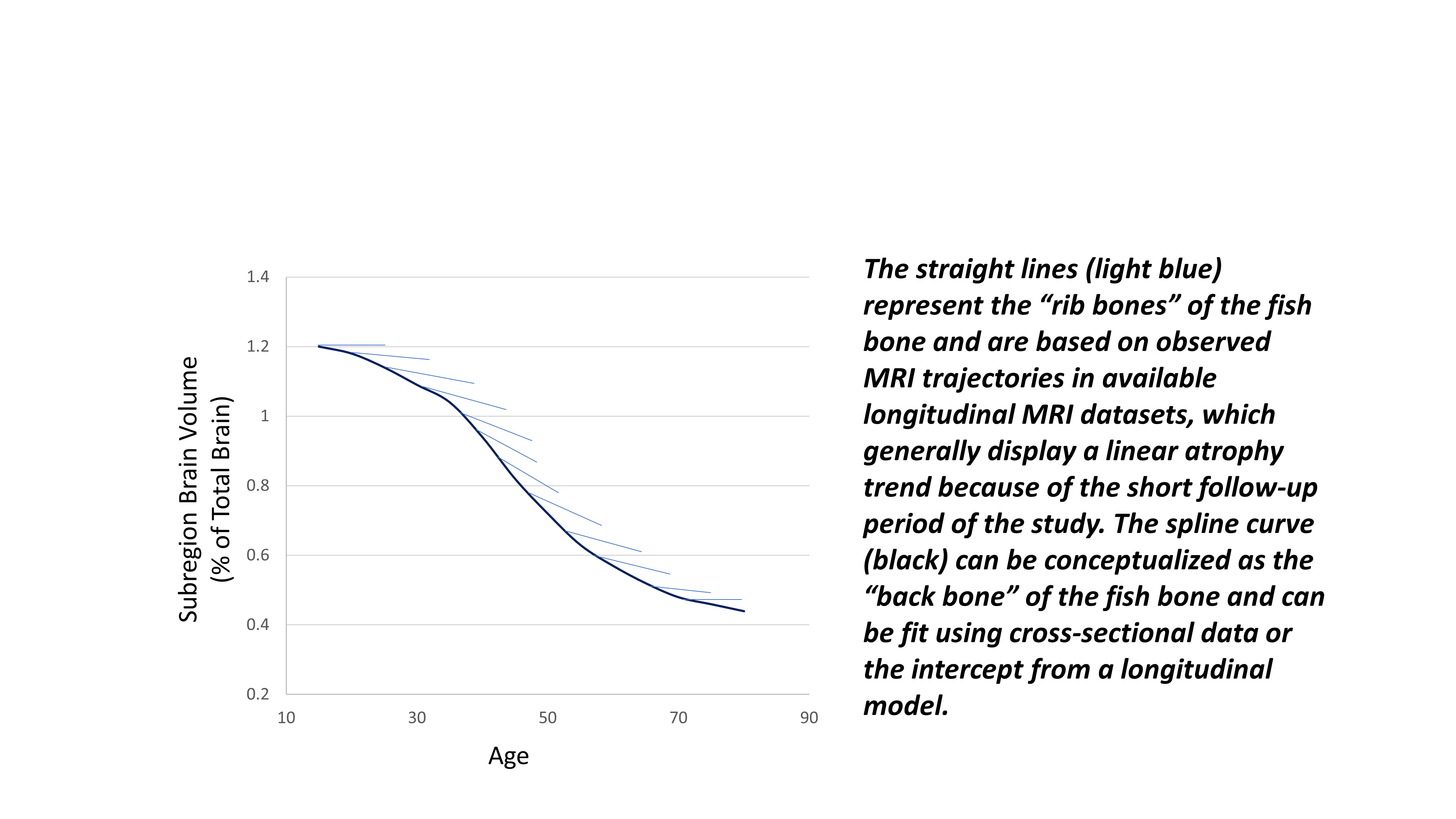

Supplement: Supplementary file 2 — Supplementary Figures. [file 41598_2023_43618_MOESM2_ESM.zip › Figure 1 43618.bmp]

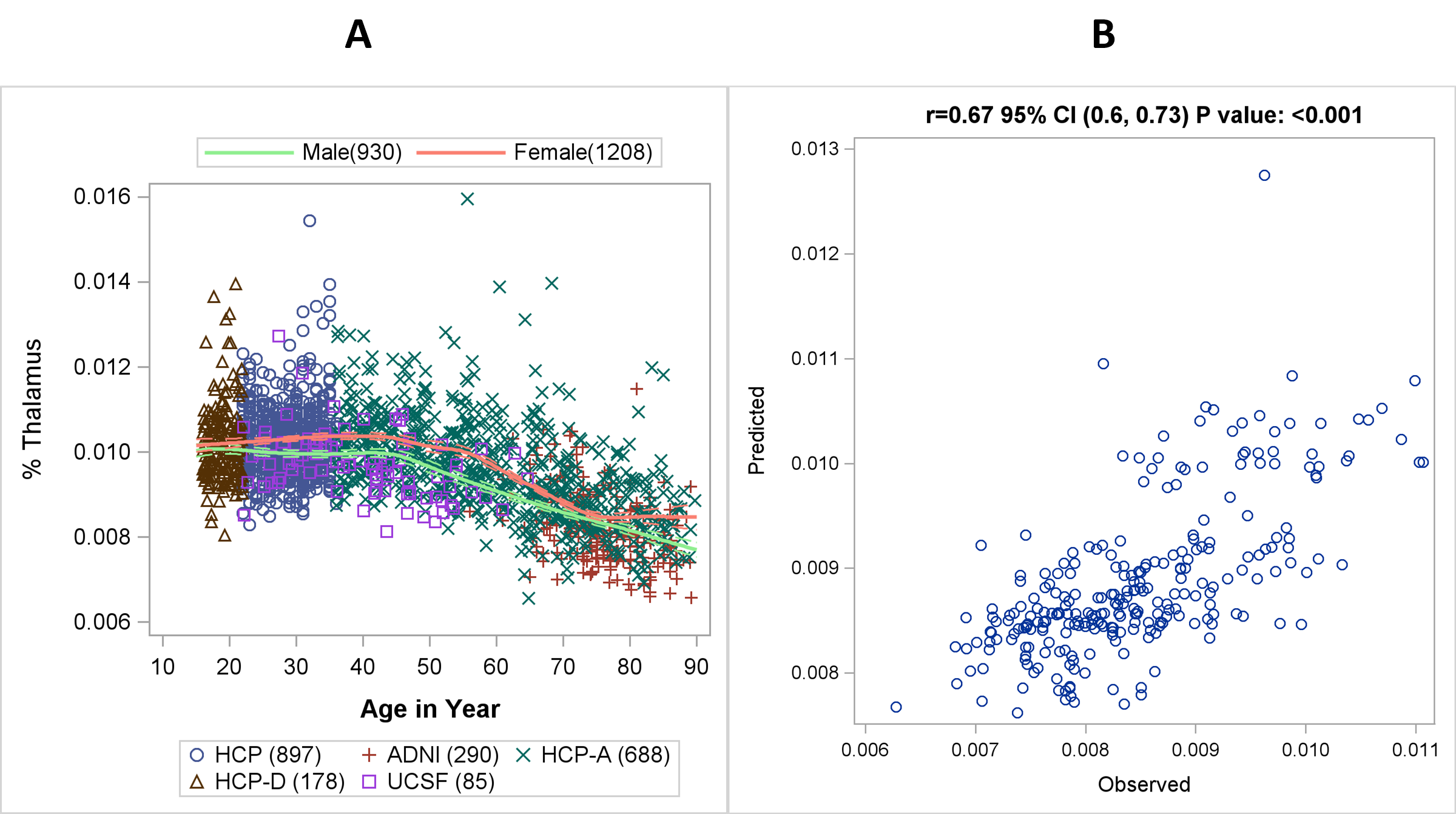

Supplement: Supplementary file 2 — Supplementary Figures. [file 41598_2023_43618_MOESM2_ESM.zip › Figure 2 43618.tif]

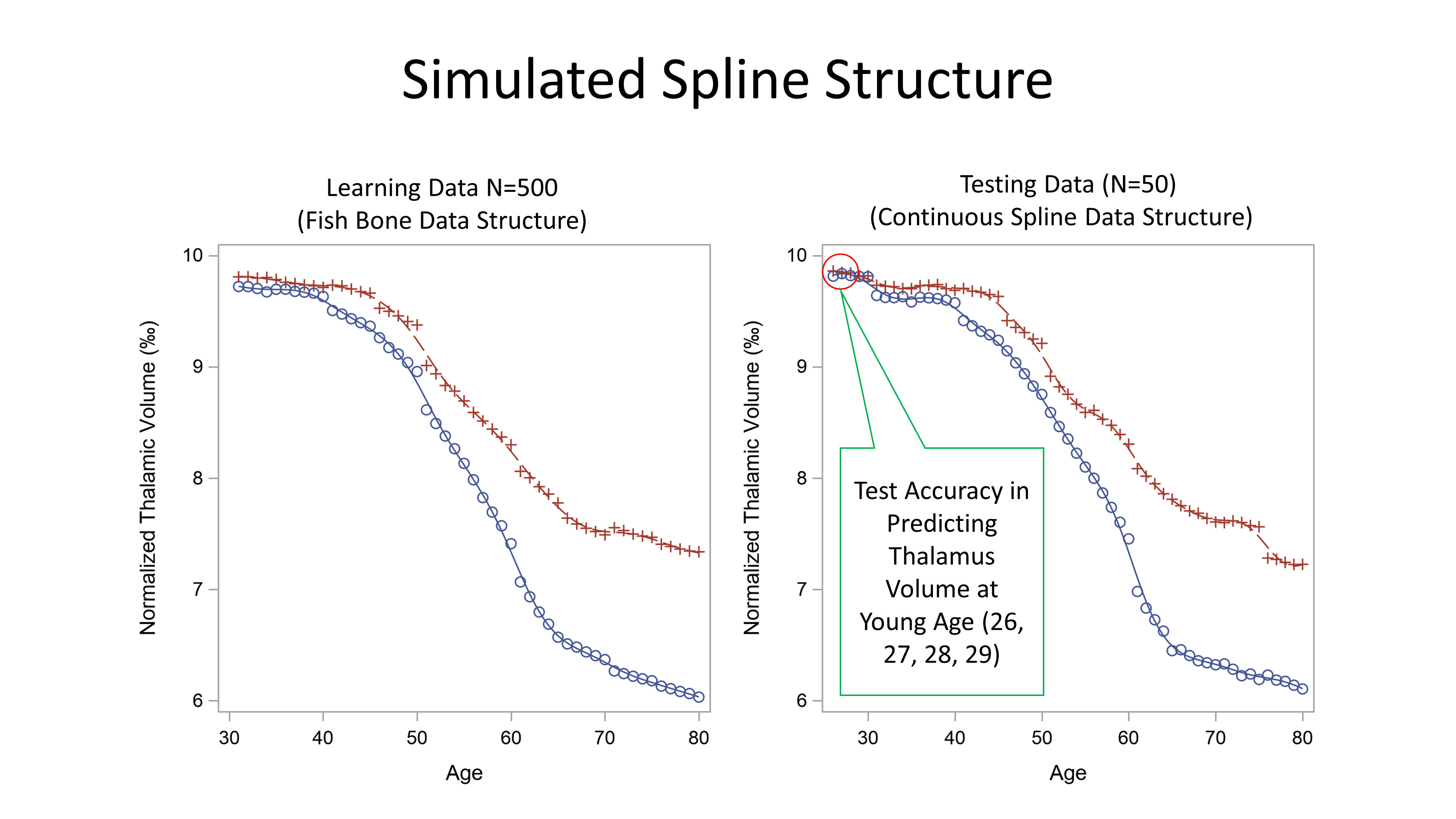

Supplement: Supplementary file 2 — Supplementary Figures. [file 41598_2023_43618_MOESM2_ESM.zip › Figure 3 43618.bmp]

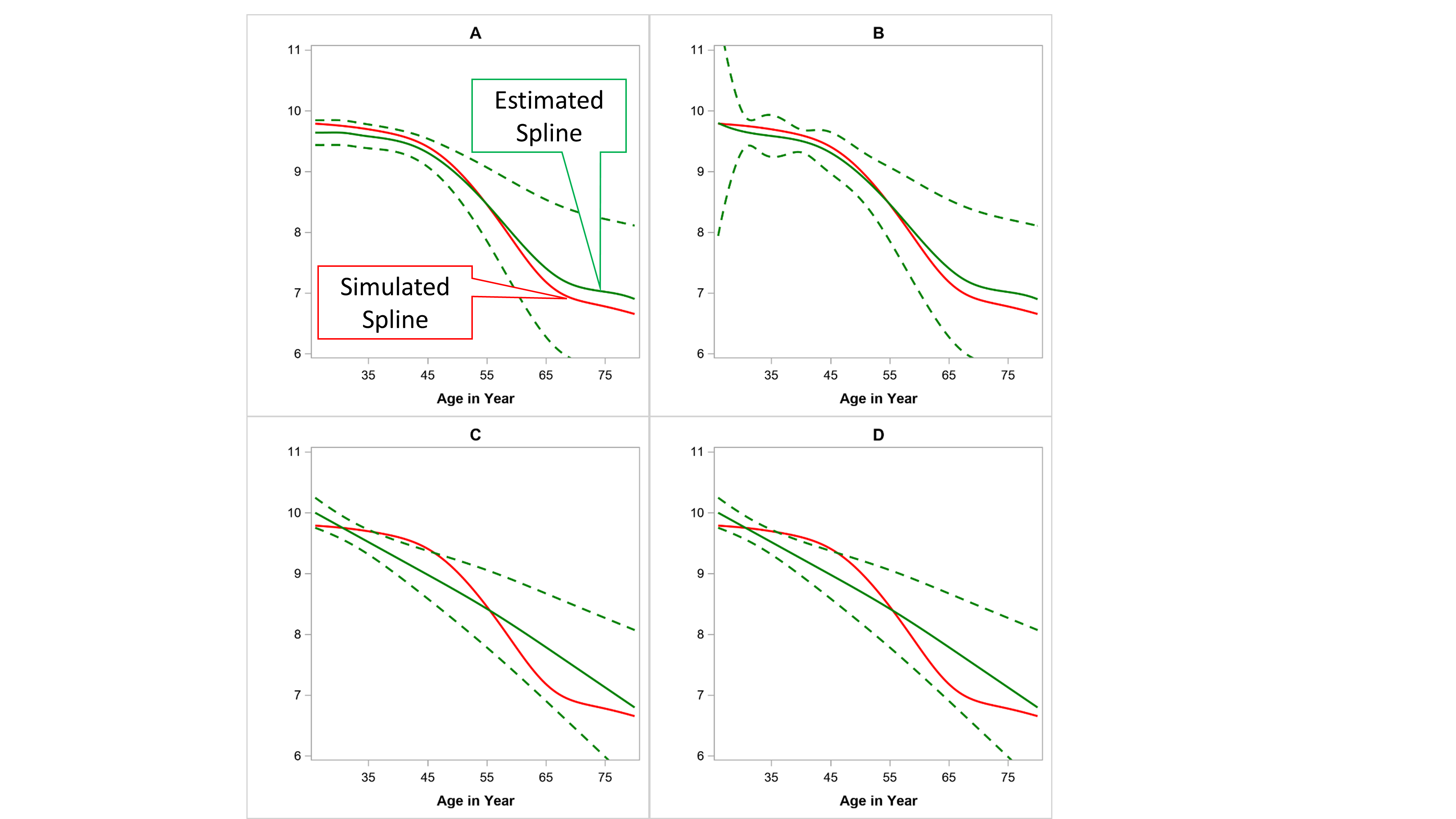

Supplement: Supplementary file 2 — Supplementary Figures. [file 41598_2023_43618_MOESM2_ESM.zip › Figure 4 43618.bmp]

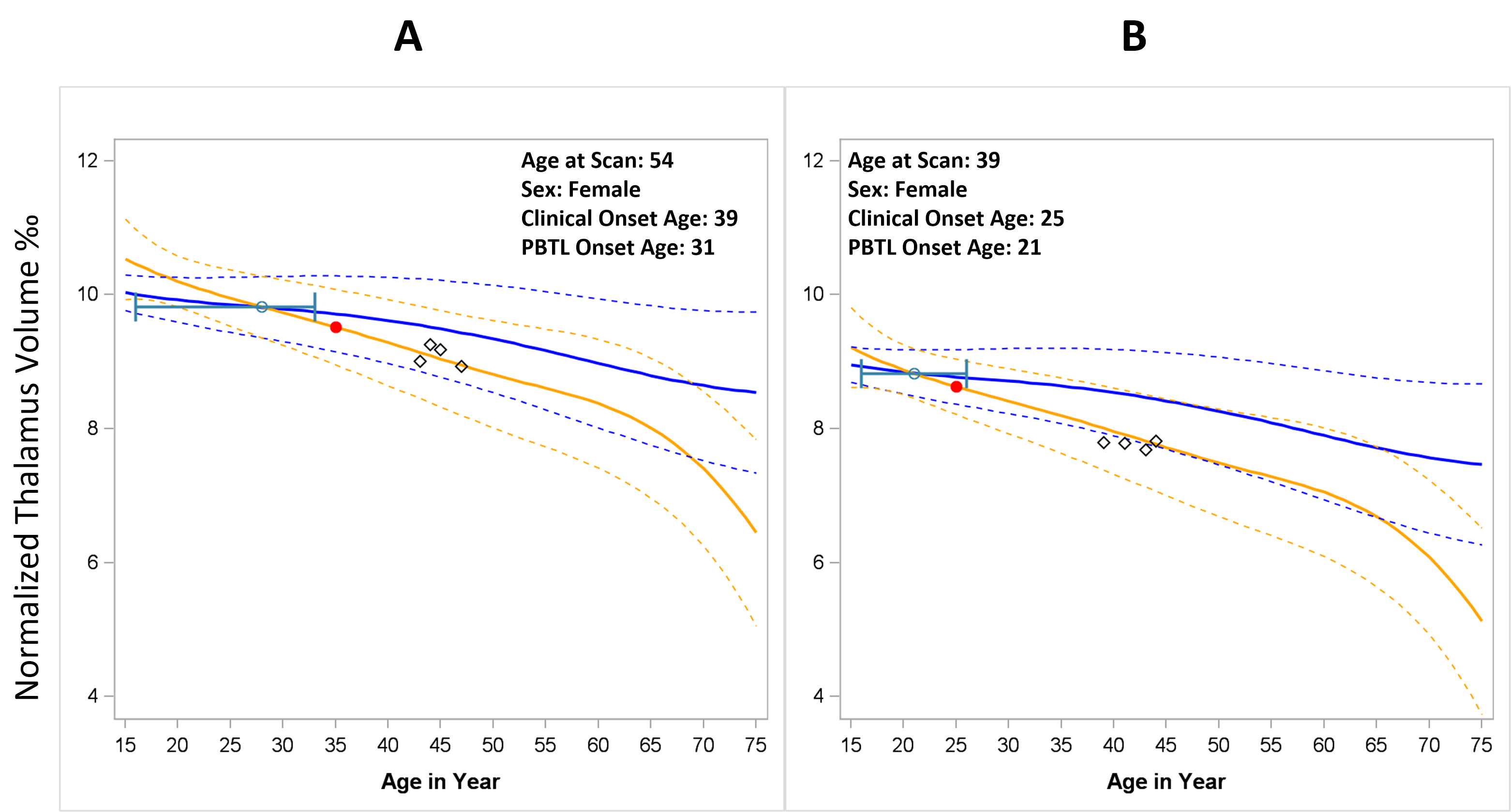

Supplement: Supplementary file 2 — Supplementary Figures. [file 41598_2023_43618_MOESM2_ESM.zip › Figure 5 43618.tif]

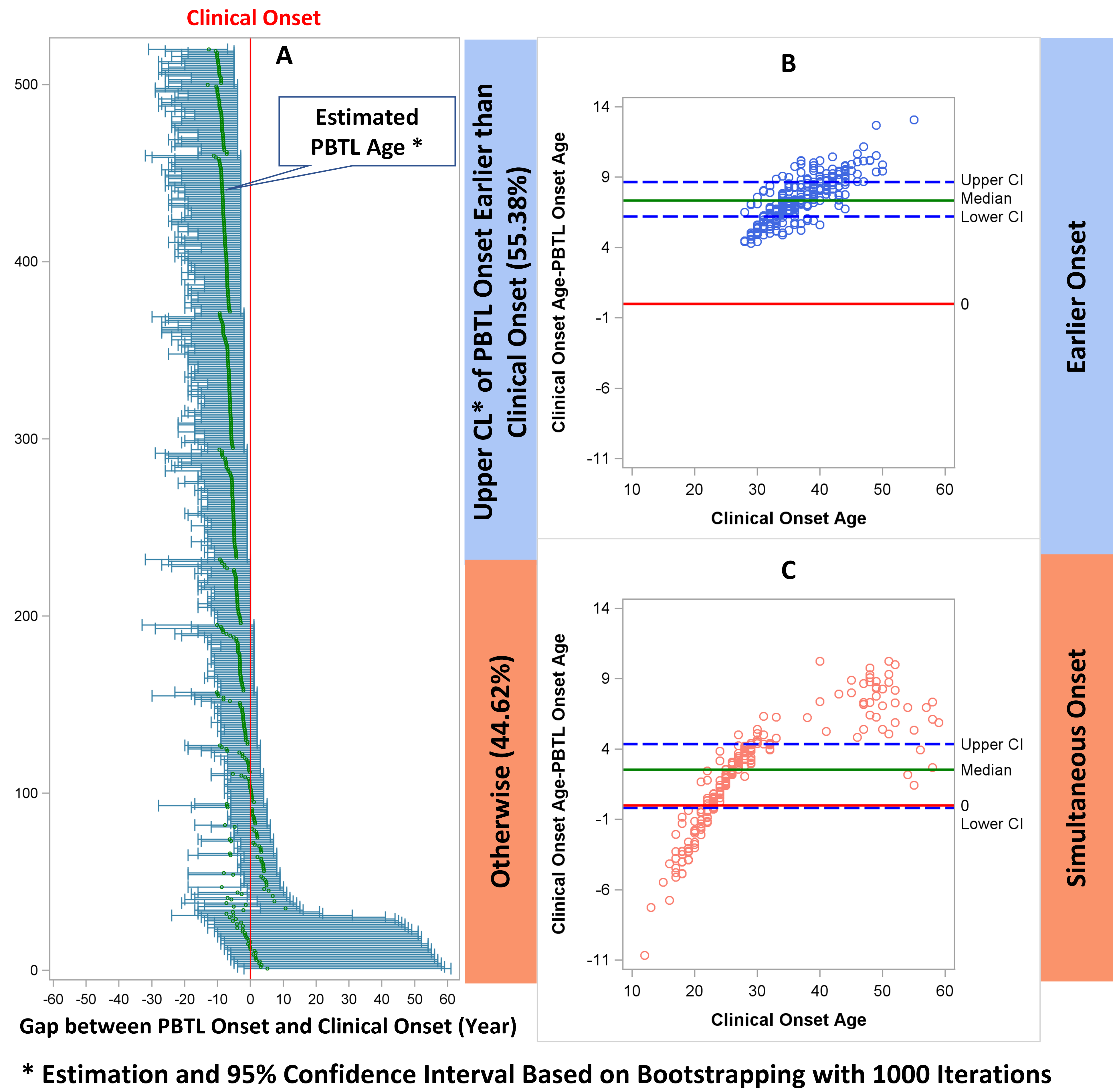

Supplement: Supplementary file 2 — Supplementary Figures. [file 41598_2023_43618_MOESM2_ESM.zip › Figure 6 43618.tif]
